# T-cell and antibody responses to first BNT162b2 vaccine dose in previously infected and SARS-CoV-2-naive UK health-care workers: a multicentre prospective cohort study

**DOI:** 10.1016/S2666-5247(21)00275-5

**Published:** 2022-01

**Authors:** Adrienn Angyal, Stephanie Longet, Shona C Moore, Rebecca P Payne, Adam Harding, Tom Tipton, Patpong Rongkard, Mohammad Ali, Luisa M Hering, Naomi Meardon, James Austin, Rebecca Brown, Donal Skelly, Natalie Gillson, Sue L Dobson, Andrew Cross, Gurjinder Sandhar, Jonathan A Kilby, Jessica K Tyerman, Alexander R Nicols, Jarmila S Spegarova, Hema Mehta, Hailey Hornsby, Rachel Whitham, Christopher P Conlon, Katie Jeffery, Philip Goulder, John Frater, Christina Dold, Matthew Pace, Ane Ogbe, Helen Brown, M Azim Ansari, Emily Adland, Anthony Brown, Meera Chand, Adrian Shields, Philippa C Matthews, Susan Hopkins, Victoria Hall, William James, Sarah L Rowland-Jones, Paul Klenerman, Susanna Dunachie, Alex Richter, Christopher J A Duncan, Eleanor Barnes, Miles Carroll, Lance Turtle, Thushan I de Silva, PITCH Consortium, Adam Harding, Adam Watson, Adrian Shields, Adrienn Angyal, Ahmed Alhussni, Alex Richter, Alexander Nicols, Alexandra Deeks, Alice Webb-Bridges, Andrew Cross, Ane Ogbe, Anni Jämsén, Anthony Brown, Anu Chawla, Christina Dold, Christopher Duncan, Christopher Conlon, Donal Skelly, Denise O'Donnell, Eleanor Barnes, Emily Adland, Esme Weeks, Gurjinder Sandhar, Hailey Hornsby, Helen Brown, Hema Mehta, Hibatullah Abuelgasim, Huiyuan Xiao, James Austin, Jarmila Spegarova, Jennifer Holmes, Jenny Haworth, Jessica Tyerman, John Frater, Jonathan Kilby, Joseph Cutteridge, Katie Jeffery, Katy Lillie, Lance Turtle, Leigh Romaniuk, Lucy Denly, Luisa Hering, M. Azim Ansari, Matthew Pace, Meera Chand, Miles Carroll, Mohammad Ali, Mwila Kasanyinga, Naomi Meardon, Natalie Gillson, Patpong Rongkard, Paul Klenerman, Philip Goulder, Philippa Matthews, Rachel Whitham, Rebecca Brown, Rebecca Payne, Robert Wilson, Sarah Rowland-Jones, Sarah Thomas, Shona Moore, Siobhan Gardiner, Stephanie Longet, Stephanie Tucker, Sue Dobson, Susan Hopkins, Susanna Dunachie, Syed Adlou, Thushan de Silva, Tom Tipton, Victoria Hall, William James, Allan Lawrie, Nikki Smith, Helena Turton, Amira Zawia, Martin Bayley, Alex Fairman, Kate Harrington, Rosemary Kirk, Louise Marsh, Lisa Watson, Steven Wood, Benjamin Diffey, Chris Jones, Lauren Lett, Gareth Platt, Krishanthi Subramaniam, Daniel Wootton, Brendan Payne, Sophie Hambleton, Sinead Kelly, Judith Marston, Sonia Poolan, Dianne Turner, Muzlifah Haniffa, Emily Stephenson, Sandra Adele, Hossain Delowar Akhter, Senthil Chinnakannan, Catherine de Lara, Timothy Donnison, Carl-Philipp Hackstein, Lian Lee, Nicholas Lim, Tom Malone, Eloise Phillips, Narayan Ramamurthy, Nichola Robinson, Oliver Sampson, David Eyre, Beatrice Simmons, Lizzie Stafford, Alexander Mentzer, Ali Amini, Carolina Arancibia-Cárcamo, Nicholas Provine, Simon Travis, Stavros Dimitriadis, Sile Johnson, Sarah Foulkes, Jameel Khawam, Edgar Wellington, Javier Gilbert-Jaramillo, Michael Knight, Maeva Dupont, Emily Horner, James Thaventhiran, Jeremy Chalk

**Affiliations:** a Department of Infection, Immunity and Cardiovascular Disease, University of Sheffield, Sheffield, UK; b Wellcome Centre for Human Genetics, Nuffield Department of Medicine, University of Oxford, Oxford, UK; c Sir William Dunn School of Pathology, Division of Medical Sciences, University of Oxford, Oxford, UK; d Peter Medawar Building for Pathogen Research, Nuffield Department of Clinical Medicine, University of Oxford, Oxford, UK; d Centre For Tropical Medicine and Global Health, Nuffield Department of Clinical Medicine, University of Oxford, Oxford, UK; f Nuffield Department of Clinical Neuroscience, University of Oxford, Oxford, UK; g Peter Medawar Building for Pathogen Research, Department of Paediatrics, University of Oxford, Oxford, UK; h Oxford Vaccine Group, Department of Paediatrics, University of Oxford, Oxford, UK; i NIHR Oxford Biomedical Research Centre, University of Oxford, Oxford, UK; j NIHR Health Protection Research Unit in Healthcare Associated Infection and Antimicrobial Resistance, University of Oxford, Oxford, UK; k Translational Gastroenterology Unit, University of Oxford, Oxford, UK; l Radcliffe Department of Medicine, University of Oxford, Oxford, UK; m Health Protection Research Unit in Emerging and Zoonotic Infections, Institute of Infection, Veterinary and Ecological Sciences, University of Liverpool, Liverpool, UK; n Translational and Clinical Research Institute Immunity and Inflammation Theme, Newcastle University, Newcastle, UK; o Mahidol-Oxford Tropical Medicine Research Unit, Bangkok, Thailand; p Sheffield Teaching Hospitals NHS Foundation Trust, Sheffield, UK; q Oxford University Hospitals NHS Foundation Trust, Oxford, UK; r Public Health England, Colindale, London, UK; s Department of Infection and Tropical Medicine, Newcastle upon Tyne Hospitals NHS Foundation Trust, Newcastle, UK; t Institute of Cancer and Genomic Science, College of Medical and Dental Science, University of Birmingham, Birmingham, UK; u University Hospitals Birmingham NHS Foundation Trust, Birmingham, UK; v Faculty of Medicine, Department of Infectious Disease, Imperial College London, London, UK; w Tropical and Infectious Disease Unit, Liverpool University Hospitals NHS Foundation Trust, member of Liverpool Health Partners, Liverpool, UK

## Abstract

**Background:**

Previous infection with SARS-CoV-2 affects the immune response to the first dose of the SARS-CoV-2 vaccine. We aimed to compare SARS-CoV-2-specific T-cell and antibody responses in health-care workers with and without previous SARS-CoV-2 infection following a single dose of the BNT162b2 (tozinameran; Pfizer–BioNTech) mRNA vaccine.

**Methods:**

We sampled health-care workers enrolled in the PITCH study across four hospital sites in the UK (Oxford, Liverpool, Newcastle, and Sheffield). All health-care workers aged 18 years or older consenting to participate in this prospective cohort study were included, with no exclusion criteria applied. Blood samples were collected where possible before vaccination and 28 (±7) days following one or two doses (given 3–4 weeks apart) of the BNT162b2 vaccine. Previous infection was determined by a documented SARS-CoV-2-positive RT-PCR result or the presence of positive anti-SARS-CoV-2 nucleocapsid antibodies. We measured spike-specific IgG antibodies and quantified T-cell responses by interferon-γ enzyme-linked immunospot assay in all participants where samples were available at the time of analysis, comparing SARS-CoV-2-naive individuals to those with previous infection.

**Findings:**

Between Dec 9, 2020, and Feb 9, 2021, 119 SARS-CoV-2-naive and 145 previously infected health-care workers received one dose, and 25 SARS-CoV-2-naive health-care workers received two doses, of the BNT162b2 vaccine. In previously infected health-care workers, the median time from previous infection to vaccination was 268 days (IQR 232–285). At 28 days (IQR 27–33) after a single dose, the spike-specific T-cell response measured in fresh peripheral blood mononuclear cells (PBMCs) was higher in previously infected (n=76) than in infection-naive (n=45) health-care workers (median 284 [IQR 150–461] *vs* 55 [IQR 24–132] spot-forming units [SFUs] per 10^6^ PBMCs; p<0·0001). With cryopreserved PBMCs, the T-cell response in previously infected individuals (n=52) after one vaccine dose was equivalent to that of infection-naive individuals (n=19) after receiving two vaccine doses (median 152 [IQR 119–275] *vs* 162 [104–258] SFUs/10^6^ PBMCs; p=1·00). Anti-spike IgG antibody responses following a single dose in 142 previously infected health-care workers (median 270 373 [IQR 203 461–535 188] antibody units [AU] per mL) were higher than in 111 infection-naive health-care workers following one dose (35 001 [17 099–55 341] AU/mL; p<0·0001) and higher than in 25 infection-naive individuals given two doses (180 904 [108 221–242 467] AU/mL; p<0·0001).

**Interpretation:**

A single dose of the BNT162b2 vaccine is likely to provide greater protection against SARS-CoV-2 infection in individuals with previous SARS-CoV-2 infection, than in SARS-CoV-2-naive individuals, including against variants of concern. Future studies should determine the additional benefit of a second dose on the magnitude and durability of immune responses in individuals vaccinated following infection, alongside evaluation of the impact of extending the interval between vaccine doses.

**Funding:**

UK Department of Health and Social Care, and UK Coronavirus Immunology Consortium.

## Introduction

Mass vaccination roll-out in some countries has allowed real-world evaluation of the effectiveness of vaccines against SARS-CoV-2. Data from Israel showed that the effectiveness of the BNT162b2 (tozinameran; Pfizer–BioNTech) mRNA vaccine 14–20 days after a single dose was 57% (95% CI 50–63) against symptomatic infection and 74% (56–86) against admission to hospital for COVID-19.^1^ The UK Government elected to increase the dosing interval of the BNT162b2 vaccine from 3 weeks to 12 weeks to prioritise rapid administration of a single dose to a greater proportion of the population. A Public Health England (PHE) study of more than 7·5 million adults aged 70 years and older showed vaccine effectiveness 28–34 days after a single BNT162b2 dose of 61% (95% CI 51–69) against symptomatic disease.^2^ The UK Office of National Statistics COVID-19 Infection Survey of PCR testing in 383 812 healthy community participants showed 66% (95% CI 60–71) vaccine effectiveness against any infection from 21 days after a single BNT162b2 dose.^3^

**Research in context**

**Evidence before this study**

We searched PubMed, MedRxiv, and BioRxiv for studies published between Jan 1, 2020, and March 13, 2021, including the search terms “T cell”, “single dose”, “SARS-CoV-2” and “vaccine”. We found a research letter from the UK reporting anti-spike T-cell and antibody responses in 72 health-care workers after a single dose of the BNT162b2 (tozinameran; Pfizer–BioNTech) vaccine, as measured by the T-SPOT Discovery SARS-CoV-2 assay (Oxford Immunotec, Oxford, UK). In 21 participants with evidence of previous SARS-CoV-2 infection, median T-cell responses were around ten times higher than in 51 infection-naive participants. Two recent papers have reported the early kinetics of the antibody and cellular response to one and two doses of the BNT162b2 vaccine, one in 11 previously infected and 11 infection-naive individuals, and another larger study. Both studies showed bigger T-cell and antibody responses after one dose in previously infected participants. A study from New York, NY, USA, of 109 individuals showed that 41 participants who were seropositive at baseline developed uniformly high spike IgG antibody responses after a single dose of either the BNT162b2 or the mRNA-1273 (Moderna) vaccine, and these levels were 10–20 times higher than responses in baseline seronegative people even after two doses. Various subsequent smaller studies have confirmed these antibody findings.

**Added value of this study**

This large, established, prospective cohort study done in 289 health-care workers across four UK National Health Service (NHS) hospitals allows parallel measurement of T-cell and antibody responses to SARS-CoV-2 vaccines, giving mechanistic insight into the vaccine effectiveness findings of the UK SIREN (SARS-CoV-2 Immunity and Reinfection Evaluation) Study in health-care workers. We show that, 28 days after a single dose of the BNT162b2 vaccine, T-cell responses were approximately five times higher in health-care workers with a previous infection than in infection-naive participants, and these levels were similar to the T-cell responses achieved after two doses in infection-naive people. Similarly, anti-spike antibody concentrations after a single dose in previously infected health-care workers were 7·7 times higher than after one dose in infection-naive health-care workers, and 1·5 times higher than after two doses in infection-naive health-care workers. Responses after a single dose in infection-naive health-care workers were equivalent to or better than those before vaccination in previously infected health-care workers. We also found that vaccination improved the breadth of T-cell responses generated in previously infected individuals and that the high antibody concentrations following a single dose in these individuals might retain neutralising activity against SARS-CoV-2 variants of concern such as the beta (B.1.351) variant.

**Implications of all the available evidence**

We provide robust evidence that, in individuals with no evidence of previous SARS-CoV-2 infection, a single dose of the vaccine generates comparable T-cell and antibody responses to those detected weeks or months after natural infection, which are highly likely to confer similar levels of protection. Two exposures to SARS-CoV-2, either by natural infection and one dose of the BNT162b2 vaccine or by two vaccine doses in infection-naive individuals, induce high levels of anti-spike T-cell responses, with higher IgG antibodies in those with previous infection than in infection-naive individuals with one or two doses of vaccine. Further work will establish whether a second dose at a 3-month interval in infection-naive individuals increases the duration and breadth of the antibody and T-cell response, giving more lasting and greater cross-protection against new viral variants of concern.

The SIREN (SARS-CoV-2 Immunity and Reinfection Evaluation) study is a large, multicentre prospective cohort study of health-care workers in UK National Health Service (NHS) hospitals.^4^ The study undertakes regular PCR and antibody screening of asymptomatic staff, and recently reported vaccine effectiveness in 23 3204 health-care workers who had received at least one dose of vaccine.^5^ At 21 days after a single BNT162b2 dose, effectiveness against symptomatic or asymptomatic infection in SARS-CoV-2 antibody-negative health-care workers was 72% (95% CI 58–86), which increased to 86% (74–96) 7 days after the second dose.

Much focus has been on the role of antibodies in vaccine-induced protection, and the role of T-cell immunity is less well characterised. The PITCH (Protective Immunity from T-cells to COVID-19 in Healthcare workers) study is a consortium of universities and PHE funded by the UK Department of Health and Social Care nested within the SIREN study that seeks to undertake immune phenotyping in health-care workers to characterise T-cell immunity induced by natural infection and vaccination. Here, we aimed to evaluate immune responses to the BNT162b2 vaccine in health-care workers, focusing on immunity induced after a single vaccine dose, and by comparing previously SARS-CoV-2-infected health-care workers with antibody-negative health-care workers.

## Methods

### Study design and participants

The PITCH study is an ongoing prospective cohort study of health-care workers recruited from five sites in the UK: the University Hospitals Birmingham NHS Foundation Trust, Liverpool University Hospitals NHS Foundation Trust, Newcastle upon Tyne Hospitals NHS Foundation Trust, Oxford University Hospitals NHS Foundation Trust, and Sheffield Teaching Hospitals NHS Foundation Trust. The present study included health-care workers from four centres (Liverpool, Newcastle, Sheffield, and Oxford). Individuals were recruited by word of mouth, hospital email communications, from hospital-based staff SARS-CoV-2 screening programmes, and when consenting for the SIREN study. All participants provided written informed consent for participation in PITCH. Eligible participants were adults aged 18 years or older, currently working as health-care workers, including allied support and laboratory staff. No exclusion criteria were applied. Recruitment was ongoing at the time of this analysis, with a planned recruitment target of 2000 health-care workers across all sites based on the feasibility and capacity of the PITCH consortium.

PITCH was recognised as a substudy of SIREN on Dec 2, 2020, and approved by the Berkshire Research Ethics Committee (REC), Health Research 250 Authority (IRAS ID 284460, REC reference 20/SC/0230). Some participants provided written informed consent for participation under other protocol-aligned REC-approved studies (see Acknowledgments section for full details). Procedures were done in compliance with the principles of the Declaration of Helsinki (2008) and the International Conference on Harmonization Good Clinical Practice guidelines.

### Procedures

Following written informed consent, up to 70 mL of blood was collected from participants before vaccination where possible and 28 (±7) days after the first BNT162b2 dose. In health-care workers who received two doses, blood samples were collected 28 (±7) days after the second dose only, due to the initial rapid roll-out of two BNT162b2 doses 3–4 weeks apart, before the dosing interval was extended to 12 weeks in the UK. Peripheral blood mononuclear cells (PBMCs) were separated from heparinised or EDTA (edetic acid) whole blood by use of density gradient centrifugation. Freshly isolated PBMCs were either used directly for interferon-γ (IFNγ) enzyme-linked immunospot (ELISpot) assays (Sheffield) or cryopreserved in liquid nitrogen for later use (Liverpool, Newcastle, and Oxford). Extracted plasma was stored at –80°C until further analysis.

IFNγ ELISpot assays were done with the Human IFNγ ELISpot Basic kit (Mabtech, Nacka Strand, Sweden; appendix 1 p 5). Overlapping peptide pools (18-mers with 10 amino acid overlap, 2 μg/mL) representing the full-length spike, membrane, or nucleocapsid SARS-CoV-2 proteins were added to 200 000–250 000 PBMCs per well. In assays with fresh PBMCs, spike peptides were divided into four pools representing positions spike_1–330_, spike_321–645_, spike_636–690_, and spike_950–1273_. In addition to comparing the magnitude of the IFNγ ELISpot response within each pool between previously infected and infection-naive health-care workers, the response distribution across pools was observed before and after vaccination in previously infected individuals. In assays of cryopreserved cells, peptides were divided into two pools to represent the spike S1 and S2 subunits. For an exploratory analysis, pools representing full-length spike proteins of seasonal human coronaviruses (HCoVs) were also included (NL63, 229E, OC43, HKU1-clade1, and HKU1-clade2) in a subset of 35 SARS-CoV-2-naive individuals with surplus PBMCs. Antigen-specific responses were expressed as spot-forming units (SFUs) per 10^6^ PBMCs after subtraction of spots in negative control wells. For assays of cryopreserved cells, a single IFNγ ELISpot protocol was agreed across the centres (appendix 1 pp 25–30). ELISpot assays were considered positive if the number of SFUs/10^6^ PBMCs was greater than the mean plus 2 SD of all the background values in the cohort assayed by the same method (fresh or cryopreserved). T-cell responses were characterised further with intracellular cytokine staining after stimulation with overlapping spike peptide pools (2 μg/mL, appendix 1 pp 6–9) in a subset of individuals with IFNγ ELISpot responses higher than 40 SFUs/10^6^ PBMCs and where additional samples were available, aiming for approximately 30 previously infected and 30 SARS-CoV-2-naive individuals (Liverpool and Sheffield cohorts). Samples were run on a BS FACSCanto II (Becton Dickinson; Franklin Lakes, NJ, USA) and the data were analysed with FlowJo (Treestar) software (appendix 1 pp 7–8). Per-cell cytokine analysis was done with Simplified Presentation of Incredibly Complex Evaluations (SPICE) software (appendix 1 p 9).

A multiplexed MesoScale Discovery immunoassay (V-PLEX COVID-19 Coronavirus Panel 3 [IgG] Kit, MesoScale Discovery, Rockville, MD, USA) was used in all individuals with available plasma samples to measure plasma IgG antibodies to SARS-CoV-2, SARS-CoV, MERS-CoV, and HCoVs, with a MULTI-SPOT 96-well, 10 Spot Plate coated (200—400 μg/mL) with three SARS-CoV-2 antigens (spike, receptor binding domain [RBD], and nucleocapsid), and spike proteins from SARS-CoV, MERS-CoV, and seasonal HCoVs OC43, HKU1, 229E, and NL63. Assays were done as per manufacturer's instructions with samples diluted 1:500 to 1:10 000 (appendix 1 pp 9–10). Antibody concentrations were quantified with a reference standard (convalescent plasma) and assigned arbitrary antibody units per mL (AU/mL). An alternative immunoassay (V-PLEX SARS-CoV-2 Panel 6 [ACE-2] Kit, MesoScale Discovery) was used to measure the ability of human plasma samples to inhibit angiotensin-converting enzyme 2 (ACE2) binding to different variants of SARS-CoV-2 spike (B lineage Wuhan-Hu-1 spike, D614G, alpha [B.1.1.7], beta [B.1.351], and gamma [P.1] variants). Assays were done as per the manufacturer's instructions with samples diluted 1:10 to 1:100 (appendix 1 pp 9–10). Individuals were defined as SARS-CoV-2 naive or previously infected on the basis of documented SARS-CoV-2-positive RT-PCR results or a positive anti-nucleocapsid IgG result from individual NHS trusts or the MesoScale Discovery assay.

Live virus neutralisation was assessed in plasma samples from the first ten previously infected and first ten SARS-CoV-2-naive individuals recruited at one site (Sheffield), by defining the plasma dilution that produces a 50% reduction in infectious focus-forming units of SARS-CoV-2 in Vero CCL81 cells in a microneutralisation assay. In brief, serial dilutions of human plasma were pre-incubated with a fixed dose of SARS-CoV-2 (B lineage VIC001 or beta), then complexes added to Vero CCL81 cells. A 1·5% carboxymethyl cellulose-containing overlay was used to prevent satellite focus formation during infection. 24 h after infection, monolayers were fixed with 4% paraformaldehyde (Merck Life Science UK, Gilingham, UK), permeabilised with 2% Triton X-100 (Merck Life Science UK) and stained for nucleocapsid (with the monoclonal antibody EY2A) or spike (with the monoclonal antibody EY6A) antigens. After development with a peroxidase-conjugated antibody and TrueBlue peroxidase substrate, infectious foci were quantified by the AID ELISPOT reader.

### Outcomes

The primary outcomes were SARS-CoV-2 spike-specific IFNγ ELISpot T-cell responses and anti-spike binding antibodies 28 (±7) days following a single dose of the BNT162b2 vaccine. T-cell responses and antibody responses following a single dose were compared in previously infected and infection-naive health-care workers. As secondary outcomes, T-cell responses between previously infected health-care workers following one vaccine dose and infection-naive health-care workers following two vaccine doses were also compared, along with antibody responses in previously infected health-care workers following one dose and infection-naive health-care workers following two doses.

### Statistical analysis

All individuals who were recruited to the PITCH study with available data at the time of writing were included in the present analysis. Paired comparisons before and after vaccination were done with the Wilcoxon matched pairs signed-rank test. Unpaired comparisons across two groups were done with the Mann-Whitney test. Unpaired comparisons across multiple groups were done with the Kruskal-Wallis test with Dunn's post-test for multiple comparisons and adjusted p values displayed. Pairwise correlations in exploratory analyses were assessed with Spearman's rank-order correlation (*r*_s_), comparing: spike-specific T-cell and antibody responses; spike-specific and RBD-specific antibody responses; time from infection to first vaccine dose with T-cell and antibody responses; age at vaccination with T-cell and antibody responses; HCoV-specific pre-vaccine antibody and T-cell responses with SARS-CoV-2 spike-specific post-vaccine responses; and surrogate neutralisation antibody concentrations with live virus microneutralisation titres. Correlation coefficients were interpreted as low (*r*_s_=0·20–0·49), moderate (*r*_s_=0·50–0·69), high (*r*_s_=0·70–0·89), or very high (*r*_s_=0·90–1·00). Geometric means and 95% CIs were estimated for binding antibody concentrations. Multivariable generalised linear regression models were created to estimate the associations between antibody or T-cell response between infection-naive and previously infected individuals following one vaccine dose, while accounting for the effect of age and sex. A threshold of p values less than 0·05 was used to define a significant result. Statistical analyses were done with R, version 3.5.1, and GraphPad Prism, version 9.0.1.

### Role of the funding source

The funders had no role in study design, data collection, data analysis, data interpretation, or writing of the report.

## Results

Between Dec 9, 2020, and Feb 9, 2021, 289 health-care workers received one (n=264) or two doses (n=25) of the BNT162b2 vaccine (table). Of those receiving a single dose, 145 were previously infected and 119 were SARS-CoV-2 naive, while all 25 health-care workers receiving two doses were SARS-CoV-2 naive. The median age was 44 years (IQR 33–52), with 19 individuals aged 60 years or older, and 232 (80%) female, as seen in the parent SIREN study.^5^ The median dosing interval for health-care workers who received two doses was 25 days (IQR 22–27). In those previously infected, 106 (73%) of 145 had a documented SARS-CoV-2-positive PCR, a median of 268 days (IQR 232–285) before vaccination. An overview of assays done is detailed in appendix 1 (p 11).

**Table:** Characteristics of health-care workers included in the study

		**Infection naive, one dose (n=119)**	**Previously infected, one dose (n=145)**	**Infection naive, two doses (n=25)**
Median age, years		37 (29–48)	47 (37–54)	46 (37–54)
Sex				
	Female	94 (79%)	125 (86%)	13 (52%)
	Male	25 (21%)	20 (14%)	12 (48%)
PCR positive		..	106 (73%)	..
Asymptomatic		..	13 (9%)	..
Time from infection to vaccine, days*		..	268 (232–285)	..
Pre-vaccine sampling, days†		19 (2–45)	19 (4–45)	..
Time from first dose to sampling, days		28 (27–32)	28 (26–33)	..
Dosing interval, days		..	..	25 (22–27)
Time from second dose to sampling, days		..	..	28 (27–31)
Centre				
	Liverpool	18	10	..
	Newcastle	28	24	..
	Oxford	22	20	25
	Sheffield	51	91	..

Data are n, n (%), or median (IQR).

* Time from infection to vaccine for PCR-confirmed participants (date of PCR test missing for two of 106 participants).

† No baseline sample available for eight of 119 participants in the infection-naive one-dose group, and five of 145 participants in the previously infected one-dose group; no participants in the infection-naive two-dose group had a pre-vaccine sample available.

**Figure 1:**
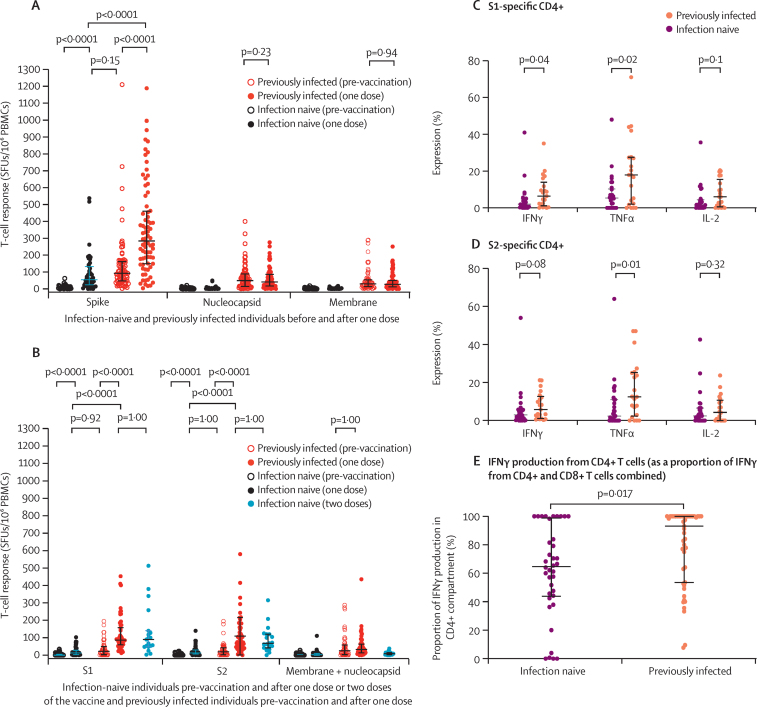
T-cell responses following BNT162b2 vaccine in SARS-CoV-2-naive and previously infected individuals (A) IFNγ ELISpot responses to a single dose in 45 infection-naive and 76 previously infected individuals (Sheffield cohort) with freshly isolated PBMCs. Summed responses from four overlapping peptide pools spanning the entire spike, nucleocapsid, and membrane protein pool responses are shown. (B) Comparison of IFNγ ELISpot responses with cryopreserved PBMCs in 58 infection-naive and 52 previously infected individuals a median of 28 days (IQR 26–33) following a single dose, along with 19 infection-naive individuals a median of 28 days (27–32) following two doses. Responses to peptide pools representing the S1 and S2 subunits of the spike protein, and a combined pool presenting membrane and nucleocapsid proteins, are shown. Pooled data from the Liverpool, Newcastle, and Oxford cohorts are shown, with centre-stratified data shown in appendix 1 (p 11). (C–E) Intracellular cytokine staining in individuals who received one dose of vaccine. Expression levels of IFNγ, IL-2, and TNFα in CD4+ T cells of 31 previously infected and 32 infection-naive individuals to the S1 protein (C) and S2 protein (D). (E) The proportion of IFNγ production from CD4+ T cells, calculated by dividing the proportion of IFNγ-positive CD4+ T cells by the total IFNγ-positive CD4+ and CD8+ T cells. Bars represent medians and IQRs. ELISpot=enzyme-linked immunospot. IFNγ=interferon-γ. IL-2=interleukin-2. PBMCs=peripheral blood mononuclear cells. SFUs=spot-forming units. TNFα=tumour necrosis factor-α.

Using freshly isolated PBMCs in IFNγ ELISpot assays (Sheffield cohort), induction of total spike-specific T-cell responses was detected in 45 SARS-CoV-2-naive individuals after a single dose (median 55 [IQR 24–132] SFUs/10^6^ PBMCs), to equivalent levels in 76 previously infected health-care workers before vaccination (93 [48–161] SFUs/10^6^ PBMCs, p=0·15; figure 1A). In these 76 previously infected individuals, a single dose resulted in spike-specific T-cell responses 5·2 times higher than those in the 45 infection-naive individuals (median 284 [IQR 150–461] SFUs/10^6^ PBMCs, p<0·0001). No changes in T-cell responses to nucleocapsid and membrane proteins were observed in either group (figure 1A). These findings were confirmed by pooled data from other centres with cryopreserved PBMCs, with alignment of results between laboratories (figure 1B; appendix 1 p 12). Total spike-specific T-cell responses (sum of S1 and S2 subunit responses) in 19 SARS-CoV-2-naive participants after two BNT162b2 doses (median 162 [IQR 104–258] SFUs/10^6^ PBMCs) given according to the licensed dosing interval were equivalent to those after a single dose in 52 previously infected individuals (median 152 [119–275] SFUs/10^6^ PBMCs, p=1·00; figure 1B). A generalised linear regression model confirmed these findings in the entire cohort with T-cell data (128 previously infected and 103 infection-naive health-care workers), accounting for the potential effects of age, sex, and fresh versus cryopreserved ELISpot assay differences. Previously infected individuals had higher spike-specific T-cell responses than infection-naive individuals following vaccination (0·77 [log_10_] SFUs/10^6^ PBMCs, p<0·0001; appendix 1 p 13). No effect of age or sex was seen, with cryopreserved ELISpots showing lower SFUs/10^6^ PBMCs than fresh assays (–0·21 [log_10_], p=0·0032; appendix 1 p 13).

To characterise spike-specific T cells further, intracellular cytokine staining was done on ELISpot-positive post-vaccine samples in a subset of 32 infection-naive and 31 previously infected single-dose vaccine recipients. Spike S1 subunit-specific IFNγ and tumour necrosis factor (TNF) production and S2-specific TNF responses in CD4+ T cells were higher in previously infected individuals than in infection-naive individuals (figure 1C, D). Higher S1-specific TNF and interleukin-2 (IL-2) production was seen in CD8+ T cells (appendix 1 p 13). In most participants, the majority of IFNγ was produced by CD4+ T cells, with the post-vaccine CD4:CD8 IFNγ ratio higher in previously infected health-care workers than in those who were SARS-CoV-2 naive (figure 1E). Effector function expression analysed on a per-cell basis showed an overall similar profile of functionality in both CD4+ and CD8+ T cells between groups (appendix 1 p 14).

**Figure 2:**
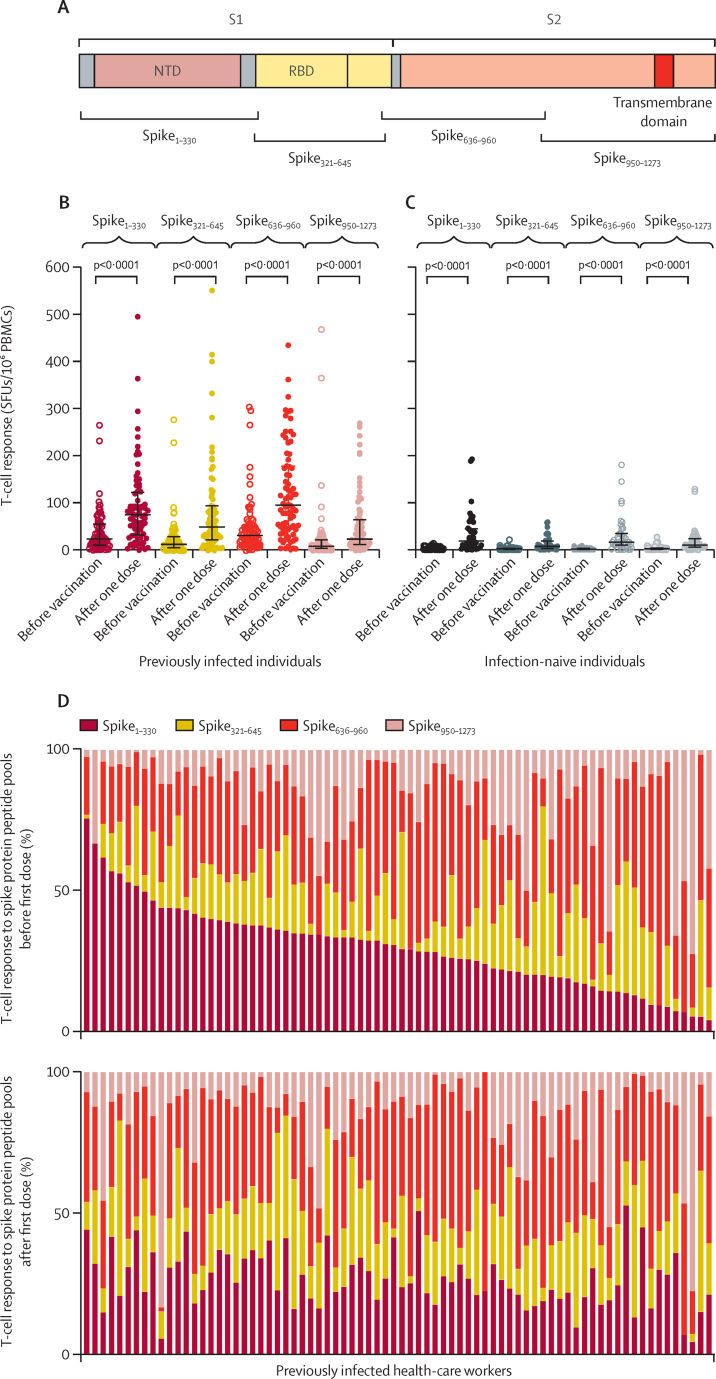
T-cell responses across spike protein following BNT162b2 vaccine with IFNγ ELISpot (A) Representation of spike protein showing positions of overlapping peptide pools used in T-cell assays in relation to S1 and S2 subunits of the spike protein, NTD, RBD, and transmembrane domain. (B, C) IFNγ ELISpot responses to a single dose in 76 previously infected (B) and 45 infection-naive individuals (C) with freshly isolated PBMCs, stratified as responses to peptide pools representing amino acids 1–330, 321–645, 636–960, and 950–1273 of the spike protein. Bars represent medians and IQRs. (D) Changes in the contribution from different peptide pools to the total spike T-cell response before and after a single dose in 76 previously infected individuals. Each column represents responses from a single individual, with responses from the same individual before and after the first dose aligned in the two plots. ELISpot=enzyme-linked immunospot. IFNγ=interferon-γ. NTD=N-terminal domain. PBMCs=peripheral blood mononuclear cells. RBD=receptor binding domain. SFUs=spot-forming units.

Vaccine-induced T-cell responses to four separate peptide pools spanning the breadth of the spike protein (spike_1–330_, spike_321–645_, spike_636–690_, and spike_950–1273_; figure 2A) were seen in both previously infected and infection-naive individuals (figure 2B, C). Responses in individuals with previous infection were characterised by the dominance of responses to spike_1–330_ and spike_636–690_ in some individuals, compared to a more balanced response across all pools in others (figure 2D). Following a single BNT162b2 dose, responses shifted to a more uniform distribution across the four pools in many individuals who had responses to one or two dominant pools before vaccination (figure 2D).

**Figure 3:**
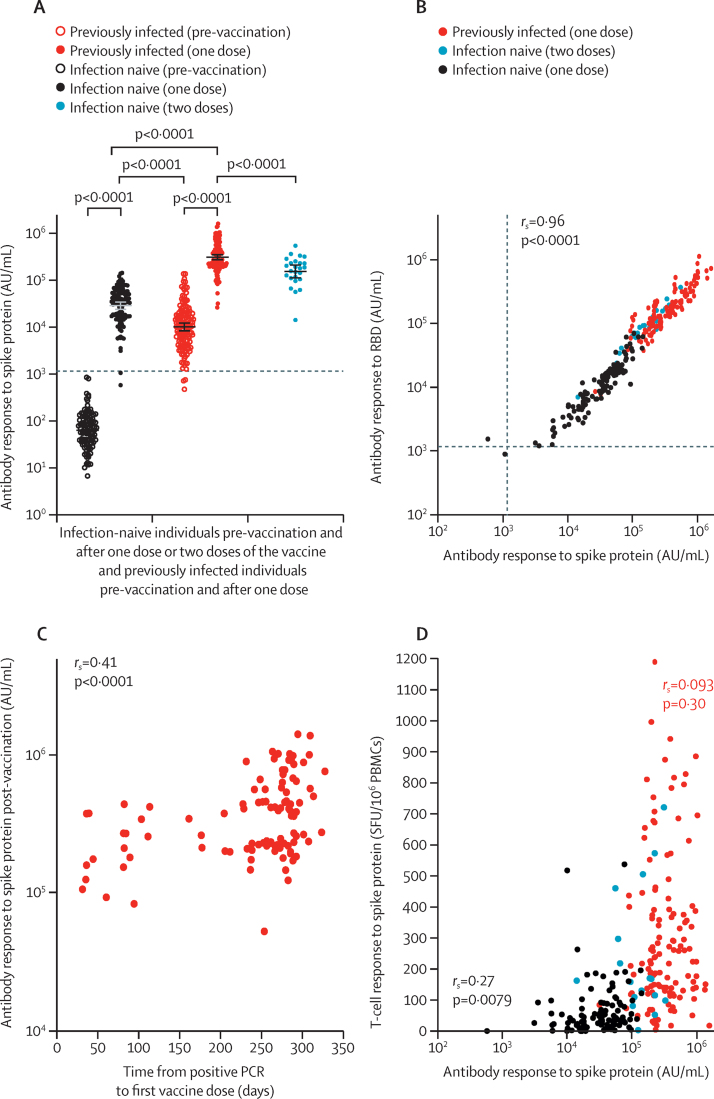
Antibody responses following BNT162b2 vaccine in infection-naive and previously infected individuals (A) Comparison of anti-spike antibody responses in 111 infection-naive and 142 previously infected individuals following a single dose and 25 infection-naive individuals following two doses. Bars represent geometric means and 95% CIs. The horizontal dotted line denotes the threshold for positivity in MesoScale Discovery SARS-CoV-2 spike assay based on mean plus 3 SD from 103 pre-pandemic negative controls (1160·3 AU/mL). Antibody titres calibrated to the WHO international standard for anti-SARS-CoV-2 immunoglobulin (National Institute for Biological Standards and Control 20/136) are shown in appendix 1 (p 14). (B) Correlation between antibody responses to the spike protein and the RBD following vaccination. Dotted lines denote the threshold for positivity in the MesoScale Discovery SARS-CoV-2 spike (1160·3 AU/mL) and RBD (1169·0 AU/mL) assay defined by pre-pandemic negative controls. (C) Relationship between time from positive SARS-CoV-2 PCR to first vaccine dose and post-vaccine anti-spike antibodies in 104 previously infected individuals. (D) Comparison of anti-spike T-cell responses measured by interferon-γ enzyme-linked immunospot and anti-spike antibodies following vaccination. Correlation coefficient (Spearman's ρ, *r*_s_) between T-cell and antibody responses following a single dose of the BNT162b2 vaccine displayed separately for 97 infection-naive and 126 previously infected individuals. Pooled responses from the entire cohort are displayed. The same data stratified by T-cell assay (fresh *vs* cryopreserved PBMCs) are shown in appendix 1 (p 17). Antibody data are presented on a log_10_ scaled axis for visualisation, with statistical comparisons done on untransformed data. AU=antibody units. PBMCs=peripheral blood mononuclear cells. RBD=receptor binding domain. SFUs=spot forming units.

Following a single BNT162b2 dose, 109 (98%) of 111 SARS-CoV-2-naive individuals mounted a spike IgG antibody response above the assay threshold to levels higher than pre-vaccine antibody concentrations in previously infected individuals (median 35 001 [IQR 17 099–55 341] AU/mL *vs* median 10 606 [IQR 4767–19 685] AU/mL, p<0·0001; figure 3A). After one dose, previously infected individuals generated antibody concentrations approximately 7·7 times higher than post-vaccine concentrations in infection-naive individuals (median 270 373 [IQR 203 461–535 188] AU/mL, p<0·0001; figure 3A). Following two doses of BNT162b2, SARS-CoV-2-naive health-care workers had median antibody concentrations of 180 904 (IQR 108 221–242 467) AU/mL, which were approximately 1·5 times lower than those following one dose in previously infected health-care workers (p<0·0001). A generalised linear regression model confirmed these findings, accounting for the potential effects of age and sex. Previously infected individuals had higher spike-specific antibody responses than infection-naive individuals following vaccination (1·05 [log_10_] AU/mL, p<0·0001; appendix 1 p 16). Increasing age was associated with lower antibody titres (–0·0070 [log_10_] AU/mL per year; p=0·0009). No effect of sex was seen. Post-vaccine antibody concentrations to the SARS-CoV-2 RBD, which is the target of many neutralising antibodies, showed a similar distribution to that of anti-spike antibodies, with a very high correlation (*r*_s_=0·96, p<0·0001; figure 3B). In previously infected individuals, a low but significant correlation (*r*_s_=0·41, p<0·0001) was seen between increasing time from SARS-CoV-2 diagnosis by PCR to first vaccine dose and post-vaccine antibody titres (figure 3C), which was confirmed in a generalised linear regression model, accounting for any effects of age and sex (appendix 1 p 16). A similar effect was not observed for spike-specific T-cell responses (appendix 1 p 17).

A low but significant positive correlation was observed between antibody and T-cell responses to a single dose of BNT162b2 in SARS-CoV-2-naive individuals (*r*_s_=0·27, p=0·0079; figure 3D). A similar low correlation was seen between antibody and T-cell responses in pre-vaccine samples from previously infected individuals (*r*_s_=0·19 p=0·035; appendix 1 p 19). However, this correlation was not seen following vaccination in previously infected individuals (*r*_s_=0·093, p=0·30; figure 3D). This loss of association appeared to be driven largely by a heterogeneous spike T-cell response to vaccination (figures 1, 3D) in the context of near-universal boosting of antibody concentrations to very high levels (figure 3A). A low inverse correlation was observed for spike antibody response and age at vaccination in the infection-naive single-dose group (*r*_s_=–0·38, p<0·0001; appendix 1 p 20), but no association between age and T-cell response was seen.

**Figure 4:**
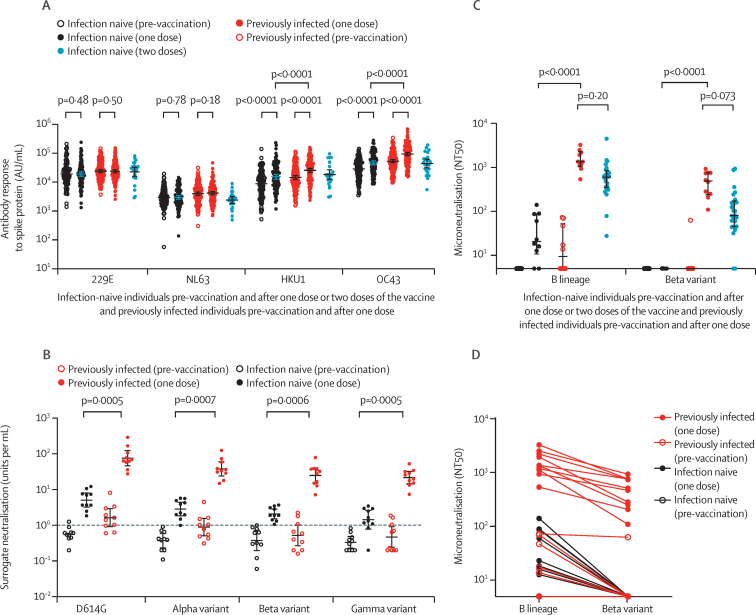
Antibody responses following BNT162b2 vaccine in naive and previously infected individuals to seasonal human coronaviruses and SARS-CoV-2 variants of concern (A) Comparison of antibody responses to spike proteins from seasonal human coronaviruses 229E, NL63, HKU1, and OC43 before and after vaccination in 111 infection-naive and 142 previously infected individuals following a single dose and 25 infection-naive individuals following two doses. (B) Surrogate neutralisation activity before and after one vaccine dose in ten naive and ten previously infected individuals to spike proteins from SARS-CoV-2 variants, including variants of concern. Activity is expressed as units per mL with 1 unit per mL equivalent to 1 μg/mL of neutralising activity of the anti-spike monoclonal antibody standard. Thresholds for positivity, based on mean plus 3 SD from 23 pre-pandemic negative control samples, were 1·02 units per mL for D614G, 1·13 units per mL for the alpha (B.1.1.7) variant, 0·93 units per mL for the beta (B.1.351) variant, and 0·98 units per mL for the gamma (P.1) variant. The horizontal dashed line denotes 1 unit per mL. (C) Ability of plasma from ten infection-naive and ten previously infected individuals, before and after a single vaccine dose, to neutralise live virus, expressed as the reciprocal titre required for 50% reduction in infectious focus-forming units (NT50) of lineage B and the beta variant of SARS-CoV-2 in a microneutralisation assay. Microneutralisation data from the 25 naive individuals sampled at 7 days following two doses have been previously described^7^ and are presented here for comparison. (D) Reduction in neutralisation titres (NT50) for each plasma sample against the beta variant compared to a B lineage virus. Antibody data are presented on a log_10_ scaled axis for visualisation, with statistical comparisons done on untransformed data. AU=antibody units.

A single BNT162b2 dose also elicited antibody responses to SARS-CoV and MERS-CoV spike trimers in both infection-naive and previously infected individuals (appendix 1 p 21), although at lower levels than those seen with SARS-CoV-2. One dose of BNT162b2 also induced antibody responses to human seasonal betacoronavirus (HKU1 and OC43) spike proteins as previously reported,^6^ but not to alphacoronavirus (229E and NL63) spike proteins, in both infection-naive and previously infected health-care workers (figure 4A). Post-vaccine antibody concentrations were higher in those with previous SARS-CoV-2 infection than in infection-naive individuals for HKU1 (median 29 666 [IQR 12 454–48 789] AU/mL *vs* 14 636 [7659–33344] AU/mL, p<0·0001) and OC43 (median 108 282 [50 278–175 835] AU/mL *vs* 48 353 [24 037–102 143] AU/mL, p<0·0001). To investigate whether previous exposure to seasonal human betacoronaviruses provides priming for antibody or T-cell responses to one BNT162b2 dose in SARS-CoV-2-naive individuals, we evaluated the relationship between pre-vaccine HKU1 and OC43 responses and post-vaccine SARS-CoV-2 spike responses in a subset of individuals. No significant correlation was seen with either antibody or T-cell responses (appendix 1 p 22).

Finally, we compared post-vaccine antibody responses to spike proteins representing D614G and the SARS-CoV-2 alpha, beta, and gamma variants of concern in a subset of ten infection-naive and ten SARS-CoV-2-infected individuals with a surrogate neutralisation assay based on competition for ACE2 binding to spike (figure 4B) and a live virus neutralisation assay (figure 4C, D). Despite equivalent pre-vaccine antibody concentrations to the beta variant in infection-naive and previously infected individuals (median 0·47 [IQR 0·25–0·81] units per mL *vs* 0·50 [0·20–1·27] units per mL), a single BNT162b2 dose resulted in a higher antibody boost in health-care workers with previous SARS-CoV-2 infection than in infection-naive health-care workers (post-vaccine median 29·7 [14·6–37·4] units per mL *vs* 1·9 [1·4–3·2] units per mL, p=0·0006). A similar pattern was observed for the other variants of concern (appendix p 24). Following a single BNT162b2 dose, no neutralising antibodies to the beta variant were observed in infection-naive individuals, with greater titres seen in previously infected health-care workers. Pre-vaccine and post-vaccine titres against the beta variant were lower for each plasma sample than for those against a lineage B SARS-CoV-2. A significant correlation was seen between surrogate and live virus neutralisation assays for both lineage B (*r*_s_=0·91, p<0·0001) and the beta variant (*r*_s_=0·78, p<0·0001; appendix 1 p 24). Neutralisation titres for the 25 infection-naive individuals sampled 7 days after two vaccine doses have been reported previously^7^ and are presented in figure 4C for comparison. These titres were lower than those in previously infected individuals following one dose, but this difference was not significant.

## Discussion

The UK Government's strategy to prioritise delivery of a first SARS-CoV-2 vaccine to certain groups while delaying the second dose^8^ elicited criticism in some quarters,^9^ but led to half the UK adult population receiving at least one dose by March 20, 2021. Here, we report the findings of a prospective study of SARS-CoV-2-specific immune responses to natural infection and vaccination in a cohort of NHS health-care workers. Antibody and T-cell responses elicited after one BNT162b2 dose in SARS-CoV-2-naive individuals were similar to or higher than pre-vaccine responses in previously infected health-care workers, although it should be noted that most of these infections were acquired during the first pandemic wave in the UK and post-infection antibody concentrations might have waned. We found that previous infection was associated with a median increase in anti-spike antibodies 7·7 times greater than those seen in SARS-CoV-2-naive individuals after the first dose. In previously infected health-care workers, a longer time interval between infection and the first vaccine dose was associated with greater boosting of antibody concentrations. One dose of BNT162b2 elicited a significant but modest increase in T-cell responses in SARS-CoV-2-naive individuals, whereas in health-care workers with previous infection, post-vaccine T-cell concentrations were approximately 5·2 times higher than in infection-naive individuals. In contrast to antibody responses, T-cell responses after two doses in infection-naive individuals were equivalent to those elicited by a single dose in previously infected participants.

Our study of 289 health-care workers builds on the findings of earlier reports describing a powerful boosting effect of previous SARS-CoV-2 infection on the antibody response to a single dose of vaccine.^10^, ^11^, ^12^, ^13^, ^14^, ^15^, ^16^ Antibody titres after two doses (given 3–4 weeks apart) in SARS-CoV-2-naive individuals did not quite reach the levels achieved after a single dose in SARS-CoV-2-primed individuals. Whether this is due to a difference between priming with infection or vaccine, or due to the duration between prime (infection) and boost (vaccine) in previously infected individuals is not known. Our finding that increasing the duration between SARS-CoV-2 infection and first vaccine dose in previously infected health-care workers was associated with greater post-vaccine antibody titres is consistent with data on continued evolution in convalescence of memory B cells able to produce broad and potent neutralising antibodies.^17^ The emergence of SARS-CoV-2 variants with spike mutations that affect antibody recognition threatens the success of SARS-CoV-2 vaccine programmes.^18^ Both the beta and gamma lineages have RBD mutations (eg, E484K) that result in reduced antibody neutralisation in vitro.^19^, ^20^, ^21^, ^22^ The efficacy of the ChAdOx1 nCoV-19 (AZD1222; Oxford–AstraZeneca) vaccine in South Africa against mild to moderate SARS-CoV-2 infection caused by the beta variant was recently shown to be as low as 10·4% (95% CI –76·8 to 54·8).^20^ Despite similar pre-vaccine antibody concentrations in a surrogate neutralisation assay against the beta and gamma variants, previously infected individuals had post-vaccine antibody concentrations approximately 15 times higher than unprimed individuals against the beta variant, and similar patterns were observed for the other variants of concern. No neutralisation of the beta variant was seen in post-vaccine plasma in infection-naive health-care workers after a single dose, whereas significantly higher titres were elicited in previously infected health-care workers. Although the antibody correlates of protection are not yet established, our data suggest that previously infected health-care workers might have greater protection against the beta variant following a single BNT162b2 dose than unprimed individuals.

Few studies have evaluated T-cell responses after one or two doses of BNT162b2. In each case, spike-specific T-cell responses were greater following vaccination in previously infected donors than in those who were SARS-CoV-2 naive.^23^, ^24^, ^25^, ^26^ In our study, an intriguing finding was that, although natural infection elicited dominant responses to just one or two of the four spike regions examined in some donors, post-vaccine responses were more balanced across all pools in most previously infected health-care workers. This finding might represent either induction of new responses against previously unrecognised regions or boosting of sub-dominant responses towards epitopes that were previously below the limits of detection. In either case, it is plausible that this broadening of the T-cell response could lead to more effective protection against emerging SARS-CoV-2 variants with antibody-escape mutations. We also found that vaccine-induced T-cell immunity following a single dose was dominated by CD4+ responses in previously infected individuals, in contrast to infection-naive participants who mounted more balanced CD4+ and CD8+ anti-spike responses. As most of the anti-spike T-cell response following natural infection is from CD4+ T cells,^27^, ^28^ this observation could also be explained by boosting of pre-existing responses below the threshold of detection, which are mostly CD4+ T cells. A sizeable fraction of the CD8+ T-cell response might be directed against non-spike proteins following natural infection, whereas in those who are naive at the time of vaccination, CD8+ T-cell responses are focused on spike perhaps as the only antigen available.

Our study has limitations worth noting. First, our cohort was predominantly female and did not evaluate vaccine responses in older people (>66 years) or in those with clinically significant health problems. It represents an evaluation of vaccine responses in a healthy UK population, which will be used to benchmark other studies in vulnerable patient groups. We accounted for the effect of age and sex in our analyses, but were not able to adjust for other potential confounders such as medical comorbidities. Although our study is one of the largest to compare T-cell responses to a single BNT162b2 dose in previously infected and SARS-CoV-2-naive individuals, we were not able to do all assays described in the whole cohort of 289 health-care workers due to feasibility or sample availability and prioritisation, or a combination of these factors. Where subsets of individuals were selected for further exploratory analyses, it is possible that unintended bias might have occurred that affected our results. As there are no precisely defined antibody or T-cell correlates of protection for SARS-CoV-2, we also cannot be certain of the degree of clinical significance of the differences we report. Equally, although we adjusted for multiple comparisons within each set of analysis, the large number of overall comparisons we report should be kept in mind when considering the relevance of statistically significant results. Finally, we do not include assessment of the delta (B.617.2) variant in our study as this variant was not yet dominant at the time of study design and execution, but would be important to evaluate in future studies.

In summary, we report detailed immunological assessments of SARS-CoV-2-specific immune responses elicited in 289 health-care workers by natural infection and vaccination with one or two doses of the BNT162b2 vaccine. In donors with no evidence of previous SARS-CoV-2 infection, a single dose of vaccine generates comparable antibody and T-cell responses to those detected weeks or months after natural infection, which are highly likely to confer similar levels of protection against infection or re-infection.^4^ Our data provide strong retrospective support for the UK policy in early 2021 of rapid roll-out of one dose of the SARS-CoV-2 vaccine to provide cover as quickly as possible for the higher-risk groups, although the effectiveness of a single dose is likely to be lower for the delta variant. Ongoing work will evaluate the extent to which previous SARS-CoV-2 infection and the vaccine dosing interval affect the duration of vaccine-induced antibody and T-cell responses.

## Data sharing

A copy of anonymised raw data used for analyses in this study has been included as appendix 2.

For more on the **PITCH study** see http://www.pitch-study.org/

## Declaration of interests

CD worked on the Oxford–AstraZeneca COVID-19 vaccine trial (phase 1–3). AO reports personal fees from Take Two Interactive and personal fees from Genome BC, outside the submitted work. PCM reports grants from the Wellcome Trust during the conduct of the study. SLR-J reports grants from the UK Department of Health and Social Care during the conduct of the study and grants from UK Research and Innovation (UKRI), National Institute for Health Research (NIHR), and Global Challenges Research Fund outside the submitted work. SD reports grants from the UK Department of Health and Social Care, UK Coronavirus Immunology Consortium (UKRI), the Huo Family Foundation, and the NIHR during the conduct of the study. CJAD reports grants from the Wellcome Trust during the conduct of the study. LT reports personal fees from Eisai outside the submitted work. All other authors declare no competing interests.
