# Supplementary material for: T-cell and antibody responses to first BNT162b2 vaccine dose in previously infected and SARS-CoV-2-naive UK health-care workers: a multicentre prospective cohort study
Source: Lancet Microbe. 2022 Jan;3(1):e21–31. doi: 10.1016/S2666-5247(21)00275-5 (PMC8577846; doi:10.1016/S2666-5247(21)00275-5)
Supplement: Supplementary appendix [file mmc1.pdf]

THE LANCET
Microbe

Supplementary appendix

This appendix formed part of the original submission and has been peer reviewed. We post it as supplied by the authors.

Supplement to: Angyal A, Longet S, Moore SC, et al. T-cell and antibody responses to first BNT162b2 vaccine dose in previously infected and SARS-CoV-2-naive UK health-care workers: a multicentre prospective cohort study. *Lancet Microbe* 2021; published online Nov 9. https://doi.org/10.1016/S2666-5247(21)00275-5.

APPENDIX

Table of Contents (page)

PITCH Consortium additional members and contributions (page 3)

Supplementary methods (pages 5 - 10)

Fig S1. Example of flow cytometry gating (pages 7 and 8)

Table S1. List of fluorochrome-conjugated antibodies used in intra-cellular cytokine staining (page 9)

Fig S2. Study profile and assays (page 11)

Fig S3. T-cell responses measured using IFNγ ELISpot assays stratified by centre (page 12)

Table S2. Factors associated with spike-specific T-cell response following a single dose of BNT162b2 in a generalised linear regression model (page 13)

Fig S4. Intra-cellular cytokine staining for spike-specific CD8+ T-cells (page 13)

Fig S5. Simplified Presentation of Incredibly Complex Evaluations (SPICE) analysis of polyfunctional T cell responses to S1 and S2 proteins (page 14)

Fig S6. Antibody responses following BNT162b2 vaccine in naïve and previously-infected individuals in WHO AU/mL (page 15)

Table S3. Factors associated with spike-specific antibody response following a single dose of BNT162b2 in a generalised linear regression model (page 16)

Table S4. Factors associated with spike-specific antibody response following a single dose of BNT162b2 in previously-infected individuals, in a generalised linear regression model (page 16)

Fig S7. Relationship between time from positive SARS-CoV-2 PCR to 1st vaccine dose and post-vaccine anti-spike T-cell response in previously-infected individuals (page 17)

Fig S8. Comparison of anti-spike T-cell responses and anti-spike antibodies (page 18)

Fig S9. Correlation between antibody and T-cell responses prior to vaccination in previously-infected individuals (page 19)

Fig S10. Correlation between age at vaccination and immune responses following BNT162b2 vaccine in naïve and previously infected individuals (page 20)

Fig S11. Antibody responses to SARS-CoV and MERS-CoV (page 21)

Fig S12. Correlation between pre-vaccine antibodies to seasonal human betacoronavirus spike protein and post-vaccine SARS-CoV-2 antibody response in naïve individuals (page 22)

Fig S13. Correlation between pre-vaccine T-cell responses to seasonal human betacoronavirus spike protein and post-vaccine SARS-CoV-2 spike T-cell responses in naïve individuals (page 23)

Fig S14. Correlation between live virus neutralisation and surrogate neutralisation assay for Wuhan-1 like (B lineage) and B.1.351/beta lineage SARS-CoV-2 (page 24)

Table S5. Surrogate neutralisation activity (units/mL) against different SARS-CoV-2 variants before and after one vaccine dose in SARS-CoV-2 naïve and SARS-CoV-2 previously infected individuals (page 24)

Standardised Operating Procedure for performing an *ex vivo* ELISpot assay using frozen peripheral blood mononuclear cells from human blood of patients (PITCH ELISpot protocol) – page 25

PITCH consortium additional members and contributions

Hibatullah Abuelgasim [32,B],
Sandra Adele[15,A],
Syed Adlou [16,E],
Hossain Delowar Akhter[15,A],
Ahmed Alhussni [32,B],
Ali Amini[23,B,C],
Carolina V. Arancibia-Cárcamo[23,22,B],
Martin Bayley[10,B],
Jeremy Chalk[26,C],
Anu Chawla [19, C] ,
Senthil Chinnakannan[15A],
Joseph Cutteridge [32,B],
Alexandra S. Deeks [7,D] ,
Catherine de Lara[15,A],
Lucy Denly [32,B] ,
Benjamin Diffey[3,B,C],
Stavros Dimitriadis[28,B,C],
Timothy Donnison[15,A],
Maeva Dupont[5,E],
David W. Eyre[22,29,B],
Alex Fairman[10,B],
Sarah Foulkes[13,B],
Siobhan Gardiner [11,B] ,
Javier Gilbert-Jaramillo[5,E],
Carl-Philipp Hackstein[15,A],
Sophie Hambleton[4,C,D],
Muzlifah Haniffa[30,29,2,17C,D],
Kate Harrington[10,B],
Jenny Haworth [17,B],
Jennifer Holmes [32,B],
Emily Horner[31,C],
Anni Jämsén, [11,B] ,
Sile Johnson[32,B],
Chris Jones[3,B,C],
Mwila Kasanyinga [16,E],
Sinead Kelly[27,B] ,
Jameel Khawam[13,B],
Rosemary Kirk[10,B],
Michael Knight[5,E],
Allan Lawrie[1,B,C],
Lian Lee[15,A],
Lauren Lett[3,B,C],
Katy Lillie [32,B],
Nicholas Lim[15,A],
Tom Malone[15, A],
Louise Marsh[10,B],
Judith Marston[27,B],
Alexander J. Mentzer[6,11,B],
Denise O'Donnell[11,C],
Brendan A I Payne[14,33,B,D],
Eloise Phillips[15,A],
Gareth Platt[3B,C],
Sonia Poolan[27,B],
Nicholas Provine[28,C],

Narayan Ramamurthy[15,C],
Nichola Robinson[15,A],
Leigh Romaniuk [17,B],
Oliver L. Sampson[15,A],
Beatrice Simmons[11,B],
Nikki Smith[1,B],
Lizzie Stafford[11,B],
Emily Stephenson[30,C,D]
Krishanthi S. Subramaniam[3,B,C],
James Thaventhiran[31,C],
Sarah Thomas [32,B],
Simon Travis[23,B],
Stephanie Tucker [17,B],
Dianne Turner[27,B],
Helena Turton[1,B],
Adam Watson [32,B],
Lisa Watson[10,B],
Alice Webb-Bridges [16,E],
Esme Weeks [32,B],
Edgar Wellington[13,B],
Robert Wilson [32,B],
Steven Wood[10,B],
Daniel G. Wootton[3,B],
Huiyuan Xiao [32, B],
Amira A.T. Zawia[1,B]

1 Department of Infection, Immunity and Cardiovascular Disease, University of Sheffield, UK
2 Wellcome Sanger Institute, Wellcome Genome Campus, UK
3 HPRU in Emerging and Zoonotic Infections, Institute of Infection, Veterinary and Ecological Sciences, University of Liverpool, UK
4 Translational and Clinical Research Institute Immunity and Inflammation Theme, Newcastle University, UK
5 Sir William Dunn School of Pathology, Division of Medical Sciences University of Oxford, UK
6 Wellcome Centre for Human Genetics, University of Oxford, UK
7 Peter Medawar Building for Pathogen Research, Nuffield Dept. of Clinical Medicine University of Oxford, UK
8 Centre For Tropical Medicine and Global Health, Nuffield Dept. of Clinical Medicine, University of Oxford, UK
9 Mahidol-Oxford Tropical Medicine Research Unit, Bangkok, Thailand
10 Sheffield Teaching Hospitals NHS Foundation Trust, Sheffield, UK
11 Oxford University Hospitals NHS Foundation Trust, Oxford UK
12 Nuffield Dept of Clinical Neuroscience, University of Oxford , UK
13 Public Health England, Colindale, London, UK
14 Department of Infection and Tropical Medicine, Newcastle upon Tyne Hospitals NHS Foundation Trust, UK
15 Peter Medawar Building for Pathogen Research, Dept of Paediatrics, University of Oxford, UK
16 Oxford Vaccine Group, Department of Paediatrics, University of Oxford, UK
17 NIHR Newcastle Biomedical Research Centre, Newcastle upon Tyne Hospitals NHS Foundation Trust, UK
18 Institute of Cancer and Genomic Science, College of Medical and Dental Science, University of Birmingham, UK

19 Tropical and Infectious Disease Unit, Liverpool University Hospitals NHS Foundation Trust, member of Liverpool Health Partners, UK
20 Faculty of Medicine, Department of Infectious Disease, Imperial College London, UK
21 NIHR Health Protection Research Unit in Healthcare Associated Infection and Antimicrobial Resistance, University of Oxford, UK
22 NIHR Oxford Biomedical Research Centre, University of Oxford, Oxford, UK
23 Translational Gastroenterology Unit, University of Oxford, UK
24 University Hospitals Birmingham NHS Foundation Trust, Birmingham, UK
25 National Infection Service, Public Health England (PHE), Porton Down, Salisbury, UK
26 Diabetes Trials Unit, Radcliffe Department of Medicine, University of Oxford, UK
27 Research Directorate, Newcastle upon Tyne Hospitals NHS Foundation Trust, UK
28 Department of Experimental Medicine, Nuffield Dept. of Clinical Medicine, University of Oxford, UK
29 Department of Dermatology, Newcastle upon Tyne Hospitals NHS Foundation Trust, UK
30 Biosciences Institute, Immunity and Inflammation Theme, Newcastle University, UK
31 MRC Toxicology Unit, University of Cambridge, UK
32 Oxford University Medical School, University of Oxford, UK
33 Translational and Clinical Research Institute, Mitochondrial and Neuromuscular Disease Theme, Newcastle University, UK

A Performed T cell experiments
B Established the clinical cohorts and collected the clinical samples and data
C Provided critical reagents, technical and intellectual expertise
D Reviewed and edited manuscript and figures
E Designed, supervised and performed antibody experiments

SUPPLEMENTARY METHODS

Interferon-gamma (IFNγ) ELISpots

IFNγ ELISpot assays were performed using the Human IFNγ ELISpot Basic kit (Mabtech, Nacka Strand, Sweden). MultiScreen-IP filter plates (Millipore, Watford, Hertfordshire, UK) were coated overnight at 4°C with the capture antibody (clone 1-D1K) at 10μg/ml. The following day, plates were blocked with RPMI media supplemented with 10% fetal bovine serum and 100 units/ml penicillin/streptomycin (R10) for two hours at 37°C. Overlapping peptide pools (18-mers with 10 amino acid overlap, Mimotopes) representing spike (S), membrane (M) or nucleocapsid (N) SARS-CoV-2 proteins were added to 200,000-250,000 PBMCs/well at a final concentration of 2μg/ml for 16–18 h. For selected individuals (cryopreserved assays only), pools representing spike proteins of seasonal coronaviruses were also included (NL63, 229E, OC43, HKU1-clade1, HKU1-clade2). Cytomegalovirus (CMV), Epstein-Barr virus (EBV) and influenza virus peptide pools (2μg/ml, Proimmune, Oxford, UK) and phytohemagglutinin (PHA, Merck Life Science UK Limited, Gilingham, UK) were used as positive controls, along with negative control wells. Wells were incubated with the biotinylated detection antibody (clone 7-B6-1, Mabtech, Nacka Strand, Sweden) at 1μg/ml for 2 hours at room temperature (RT). Streptavidin-Alkaline Phosphatase (ALP) (Mabtech, Nacka Strand, Sweden) at 1μg/ml was added for 45-90 minutes, prior to detection using the Alkaline Phosphatase (ALP) Conjugate kit (BioRad, Watford, Hertfordshire, UK). Plates were scanned and analysed with the AID Classic ELISpot reader (software version 8.0, Autoimmune Diagnostika GmbH, Germany). Antigen-specific responses were quantified by subtracting the mean spots of the control wells from the test wells and the results were expressed as spot-forming units (SFU)/10^6 PBMCs. For assays using cryopreserved cells, a single IFNγ ELISpot protocol was agreed across the centres. PBMCs were thawed and rested for 3-6 hours at 37°C prior to further use. ELISpot assays were considered positive if the

number of SFU/million PBMC was greater than the mean +2 SD of all the background values in the cohort assayed by the same method (ie fresh or cryopreserved), after background subtraction.

Intra-cellular cytokine staining protocol

1-1.5 x 10^6 cells were plated in R10 with overlapping spike peptide pools and co-stimulatory antibodies (anti-CD28, Becton Dickinson, Wokingham, Berkshire, UK and anti-CD49d, Becton Dickinson, Wokingham, Berkshire). Ionomycin (500ng/ml) and Phorbol 12-myristate 13-acetate (PMA, 50ng/ml, Merck Life Science UK Limited, Gilingham, UK) were used as positive controls. Degranulation of T-cells (a functional marker of cytotoxicity) was measured by the addition of an anti-CD107a-specific antibody (Becton Dickinson, Wokingham, Berkshire) at 1:20 dilution during the culture. Cells were incubated at 37°C, 5% CO_2 for 1 hour before adding Brefeldin A (10 μg/ml, Merck Life Science UK Limited, Gilingham, UK). Samples were incubated at 37°C, 5% CO_2 for a further 5 hours prior to staining for flow cytometry. First, stimulated cells were stained with live/dead stain 1:500 at room temperature (RT) in the dark for 20 minutes then washed in Dubelcco's Phosphate Buffered Saline (DPBS) (Invitrogen, 14190-144) by spinning the samples at 300g for 5 minutes. Cells were then fixed in 2% formaldehyde (Merck Life Science UK Limited, Gilingham, Dorset, UK, 158127) for 20 minutes before another washing step followed by permeabilisation in 1x Perm/Wash buffer (Beckton Dickinson, Wokingham, Berkshire, 554273) for 20 minutes at RT. Staining was performed in the dark at RT for 30 minutes in 1x Perm/Wash buffer with the antibodies listed in Table S1, then the cells were washed and resuspended in DPBS.

A

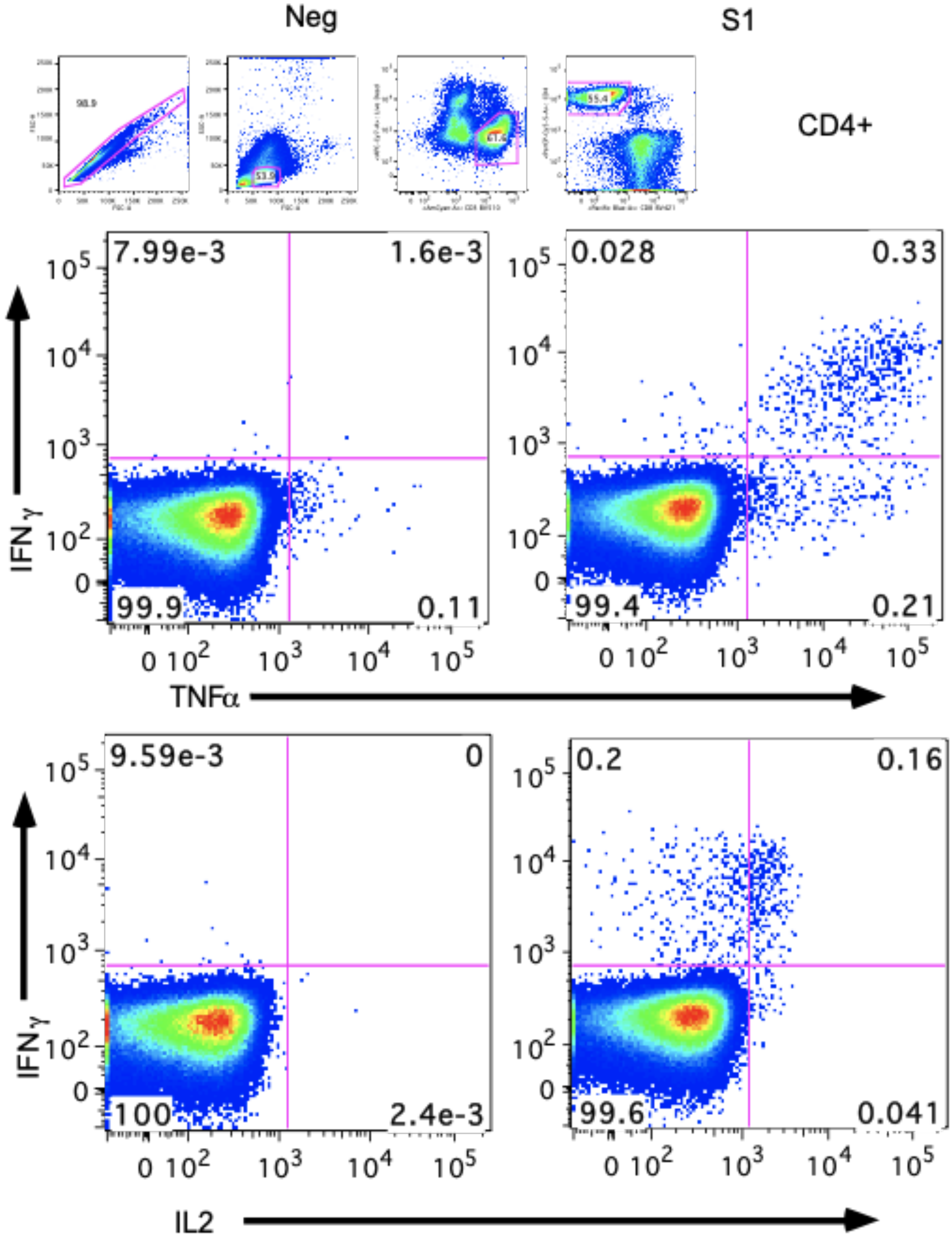
Neg
S1
CD4+
7.99e-3
1.6e-3
99.9
0.11
0.028
0.33
99.4
0.21
IFNγ
TNFα
9.59e-3
0
100
2.4e-3
0.2
0.16
99.6
0.041
IFNγ
IL2

B

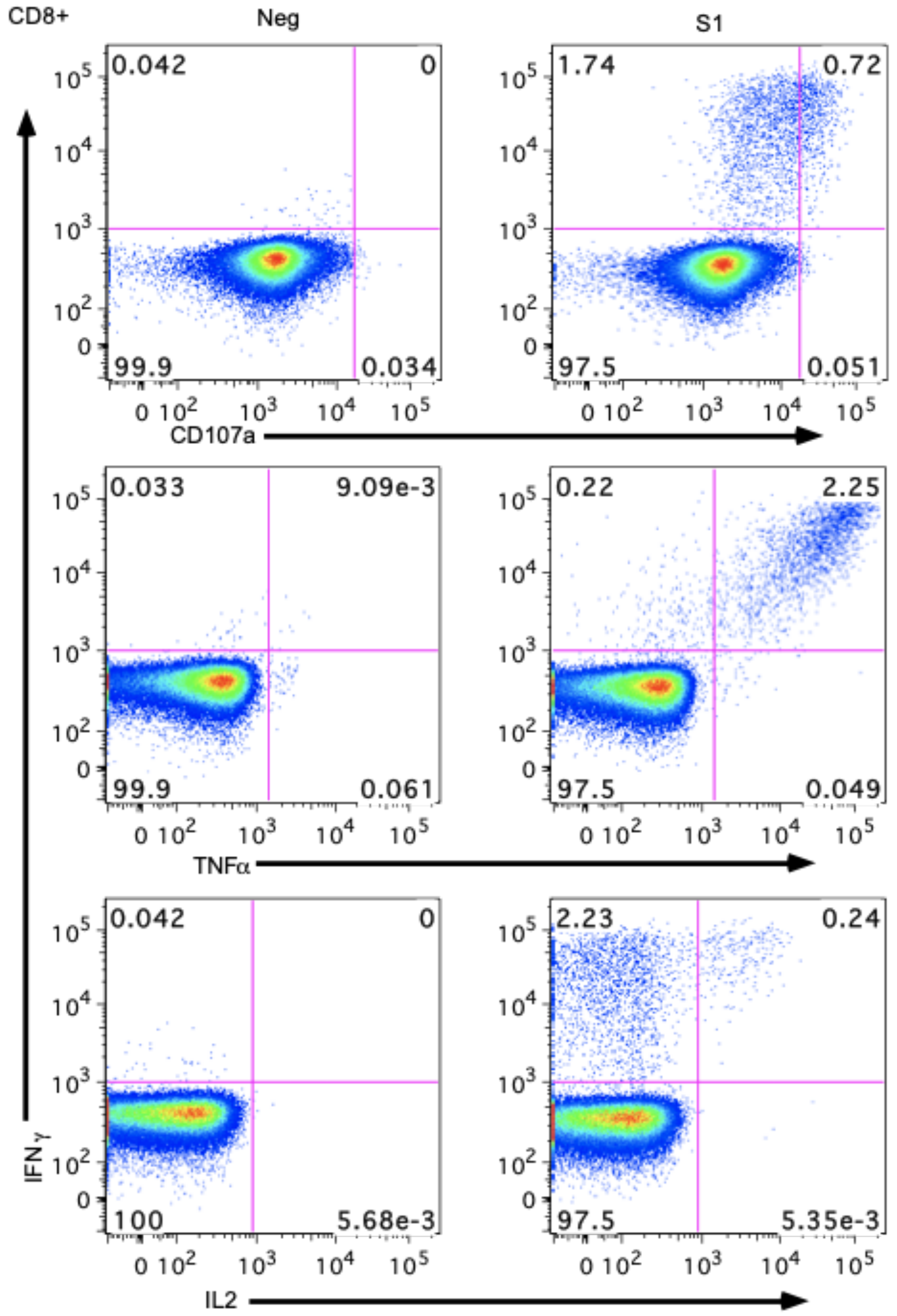

Fig S1. Example gating of CD4+ (A) and CD8+ (B) flow cytometry data from intracellular cytokine staining experiments. The gating strategy shown in (A) and the gating strategy for (B) is identical except for gating on the CD8+ rather than CD4+ population.

Table S1. List of fluorochrome-conjugated antibodies used in intra-cellular cytokine staining

Marker	Fluorochrome	Clone	Supplier	Cat. no.	dilution
CD3	BV510	UCHT1	Beckton Dickinson, Wokingham, Berkshire, UK	563109	1:50
CD4	PerCP-Cy5.5	RPA-T4	Biolegend UK Ltd., London, UK	300530	1:50
CD8	BV421	RPA-T8	Becton Dickinson, Wokingham, Berkshire, UK	562428	1:200
CD14	APC-Cy7	MφP9	Becton Dickinson, Wokingham, Berkshire, UK	557831	1:50
IFNg	FITC	45-15	Miltenyi Biotec Ltd. Bisley ,Surrey, UK	130-091-641	1:50
TNF	PE	MAb11	ThermoFisher, Warrington, UK	12-7349-82	1:100
IL-2	APC	5344.111	Becton Dickinson, Wokingham, Berkshire, UK	340450	1:25
CD107a	PE-Cy7	H4A3	Becton Dickinson, Wokingham, Berkshire, UK	561348	1:20
Cell viability dye	Near infrared	NA	ThermoFisher, Warrington, UK	L10119	1:500

SPICE analysis from intra-cellular cytokine data

Expression of multiple effector functions on a per-cell basis (“polyfunctional T cells”) was assessed using Simplified Presentation of Incredibly Complex Evaluations (SPICE) software). In order to reliably include specimens with enough events for the analysis, positive responses were defined as >0.02% IFNγ/TNF double positive cells, at least double the DMSO value, and with >50 events in any double positive gate. Data were background subtracted before importing into SPICE, and a threshold of >0.001 was applied. The difference in T-cell functionality between naive and previously infected participants was assessed using the SPICE permutation test, running 10,000 simulations.

MSD assay details (Binding IgG)

To measure IgG antibodies, 96-well plates were blocked with MSD Blocker A solution for 30 minutes. Following washing with Wash Buffer, the diluted samples (1:500-1:10000 in diluent buffer) were added to wells for 2 hours at RT alongside with the reference standard and internal controls. After another wash, wells were incubated with the detection antibody (MSD SULFO-TAG™ Anti-Human IgG Antibody, 1:200 in diluent buffer) for 1 hour at RT. Following washing, the electrochemiluminescent signal was detected by adding MSD GOLD™ Read

Buffer B and the plates were read using a MESO® SECTOR S 600 Reader. The threshold for positivity for anti-SARS-CoV-2 spike (S), receptor binding domain (RBD) and nucleocapsid (N) antibodies was determined based on the mean + 3 standard deviations from n=103 pre-pandemic controls (spike = 1160.3 AU/mL, RBD = 1169.0 AU/mL, nucleocapsid = 3874 AUmL) .

MSD assay details (ACE2 inhibition surrogate neutralisation)
To measure ACE2 inhibition, 96-well plates were blocked with MSD Blocker for 30 minutes. Plates were then washed in MSD washing buffer, and samples were diluted 1:10 – 1:100 in diluent buffer. An ACE2 calibration curve consisting of a anti-spike monoclonal antibody with equivalent activity against spike variants was used to interpolate results as units/millilitre (mL). Each unit/mL is equivalent to 1 microgram/mL of neutralising activity of monoclonal antibody standard. Furthermore, internal controls and the WHO international standard were added to each plate. After 1-hour incubation, recombinant human ACE2-SULFO-TAG™ was added to all wells. After a further 1-hour, plates were washed and MSD GOLD™ Read Buffer B was added, plates were then immediately read using a MESO® SECTOR S 600 Reader. Activity estimated by measuring the presence of antibodies able to block the binding of angiotensin-converting enzyme 2 (ACE-2) to SARS-CoV-2 spike proteins from D614G, B.1.1.7/alpha, B.1.351/beta and P.1/gamma, and expressed as units/mL. 1 unit/mL is equivalent to 1 microgram/mL of neutralising activity of the anti-spike monoclonal antibody standard. Thresholds for positivity, based on mean + 3 standard deviations from n=23 pre-pandemic negative control samples, were 1.02 units/mL for D614G, 1.13 units/mL for B.1.1.7/alpha, 0.93 units/mL for B.1.351/beta and 0.98 units/mL for P.1/gamma.

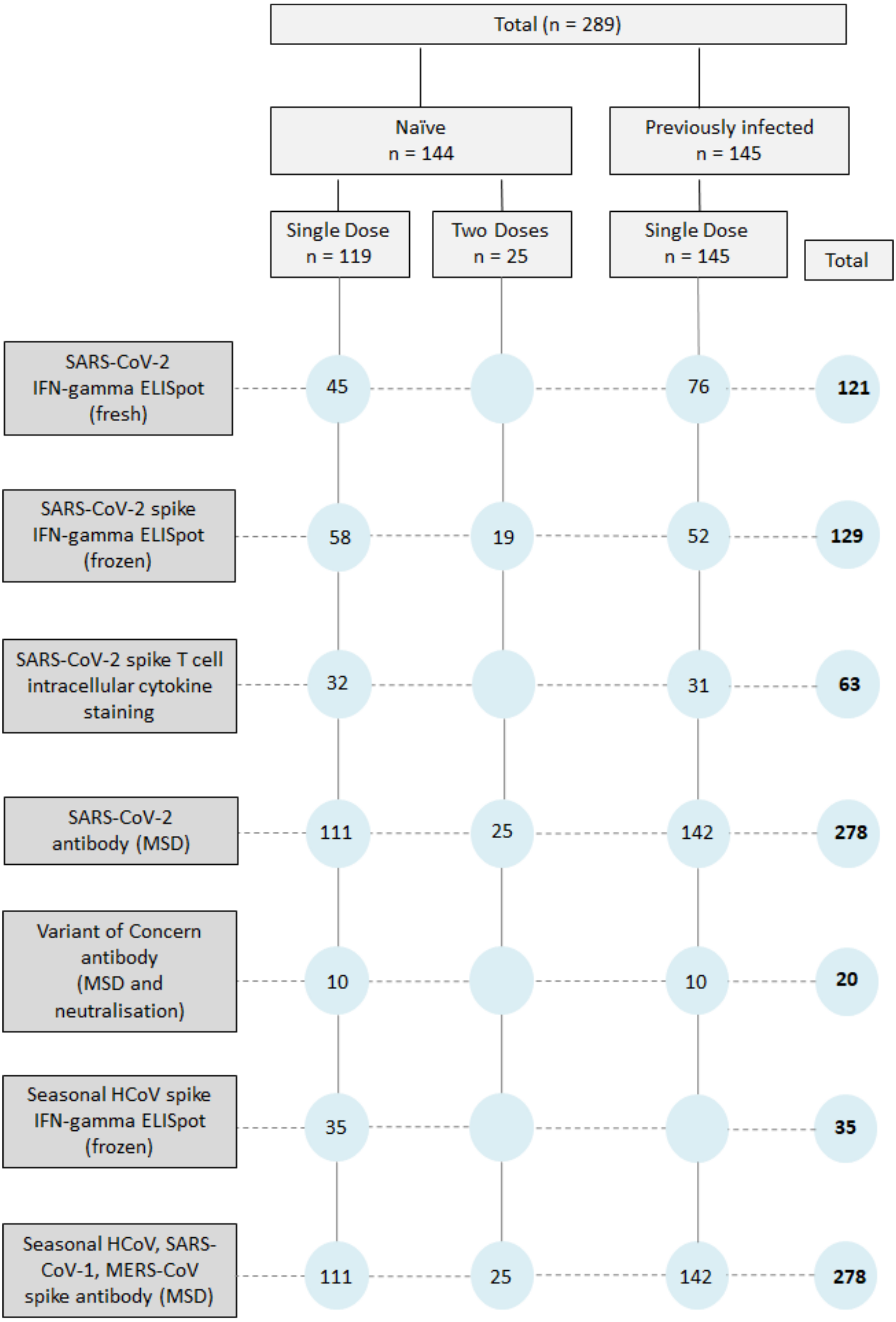

Figure S2. Study profile. *Overview of the study cohorts and assays performed to characterise the immune responses. IFN = interferon, MSD = Mesoscale Discovery, HCoV = seasonal human coronaviruses, SARS-CoV= 2003 Severe Acute Respiratory Syndrome virus, MERS-CoV = Middle Eastern Respiratory Virus.*

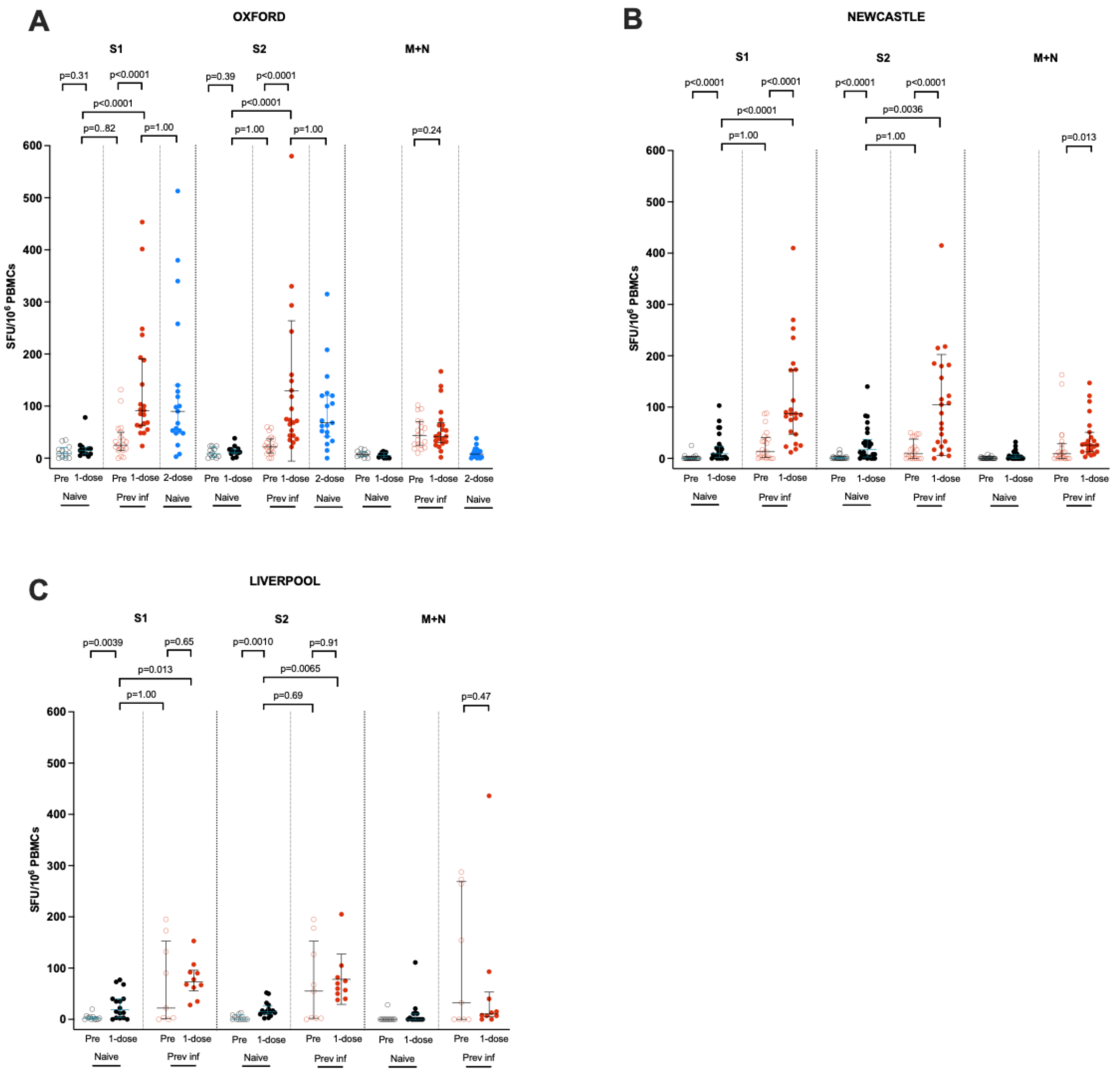

Figure S3. T-cell responses measured using IFNγ ELISpot assays stratified by centre. Participants recruited in (A) Oxford (n=55) (B) Newcastle (n=49) and (C) Liverpool (n=26). Cryopreserved peripheral blood mononuclear cells (PBMCs) used to measure responses to S1 and S2 subunits, along with nucleocapsid (N) and membrane (M) proteins using overlapping peptide pools. Values expressed as spot forming units per million PBMCs (SFU/10^6). Responses to a single vaccine dose compared between naïve (n=59) and previously-infected (n=52, Prev Inf) individuals in all three centres, along with responses to two doses in naïve individuals recruited in Oxford (2-dose, N=19). Bars represent median +/- interquartile range (IQR). Paired comparisons before and after vaccination were performed using the Wilcoxon matched pairs signed rank test. Unpaired comparisons across two groups were performed using the Mann-Whitney test. Unpaired comparisons across multiple groups were performed using the Kruskal-Wallis test with Dunn's post-test for multiple comparisons (adjusted *P* values displayed). Open circles represent responses prior to vaccination. Closed circles represent responses after vaccination.

Table S2. Factors associated with spike-specific T-cell response following a single dose of Pfizer BNT162b2 vaccine in a generalised linear regression model (n=231)

Variable	Change in spike-specific T-cell (log10 SFU/10^6 PBMCs)	Standard Error	*P* value
Previously-infected (vs naive)	+0.77	0.070	<0.0001
Age (in years)	-0.0030	0.0032	0.34
Sex	+0.061	0.089	0.50
ELISpot protocol - cryopreserved (vs fresh)	-0.21	0.070	0.0032

PBMCs = peripheral blood mononuclear cells, SFU = spot forming units

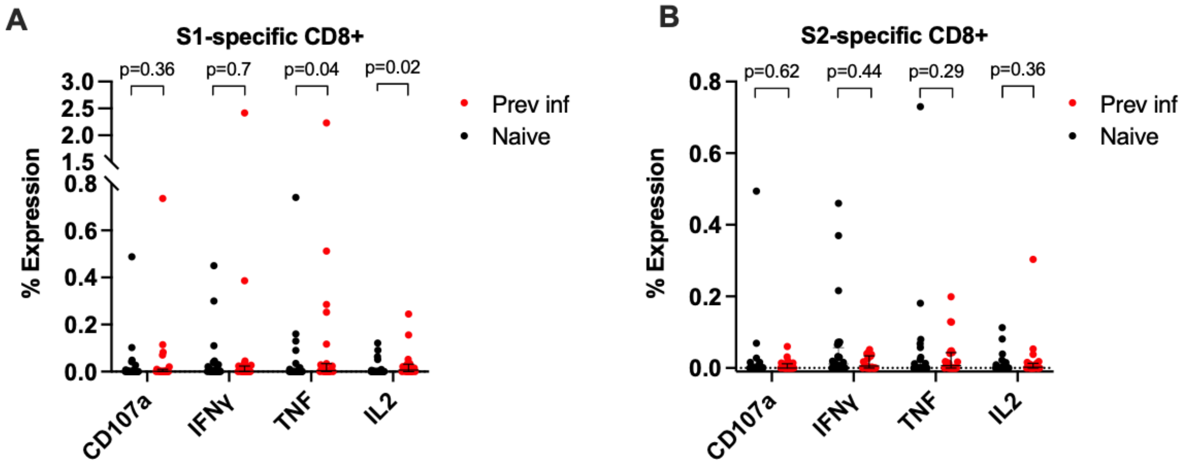

Figure S4. Intracellular cytokine staining (ICS) performed on naïve or previously-infected individuals who received one dose of vaccine and demonstrated responses in IFNγ ELISpots. Expression levels of CD107a, IFNγ, interleukin-2 (IL2), and tumour necrosis factor (TNF)α in CD8+ T cells of previously-infected (n=31, red) and naïve (n=32, black) individuals to S1 protein (**A**) and S2 protein (**B**). Bars represent median +/- IQR. Unpaired comparisons across two groups were performed using the Mann-Whitney test.

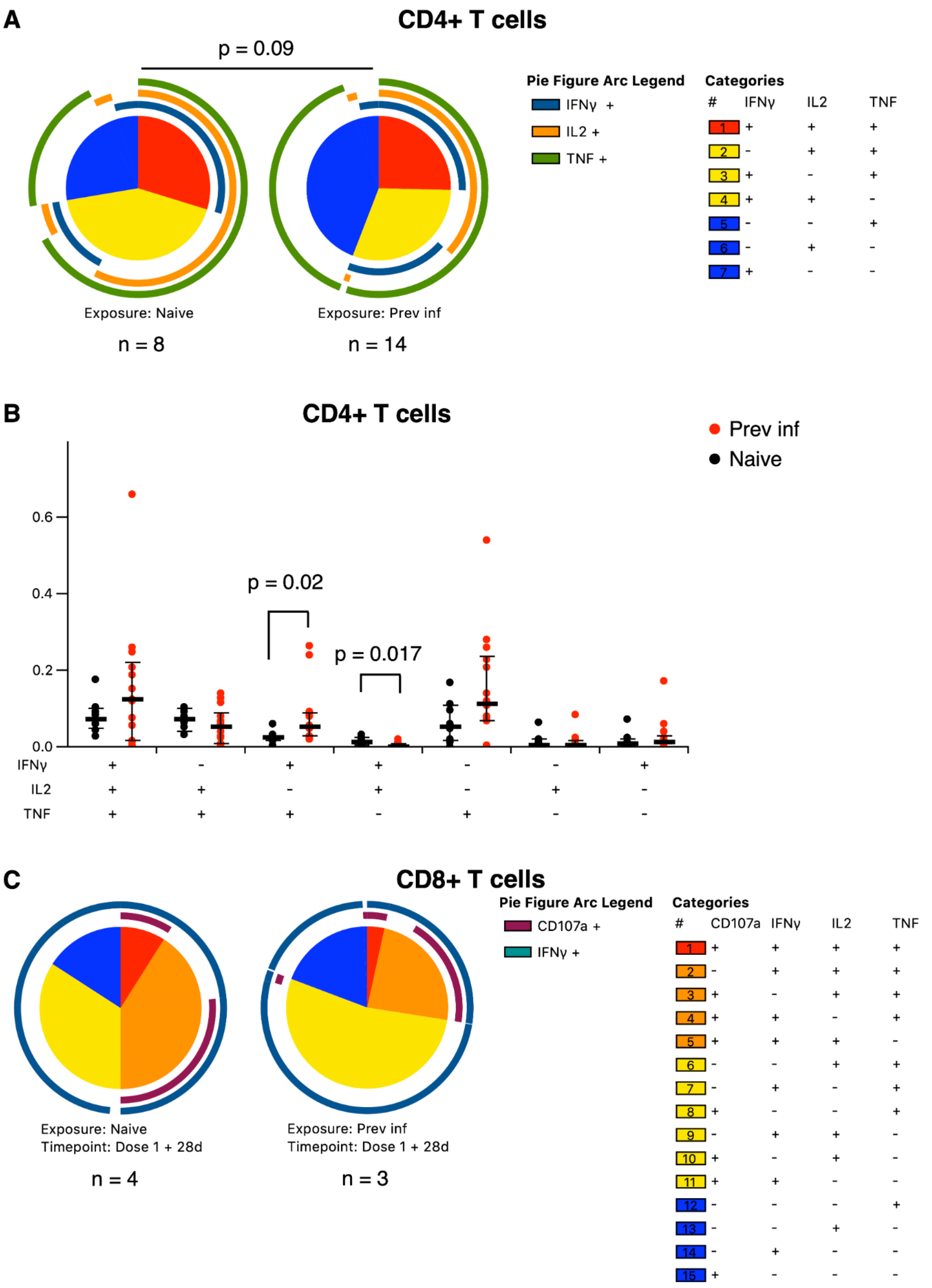

Figure S5. Simplified Presentation of Incredibly Complex Evaluations (SPICE) analysis of polyfunctional T cell responses to S1 and S2 proteins. SPICE analysis of CD4+ T cell responses (**A**, **B** n = 8 naïve and 14 previously infected) and CD8+ T cell responses (**C**, n = 4 naïve and 3 previously infected). Positive responses included in the analysis were defined as > 0.02% IFNγ/TNF double positive cells, at least double the negative control (DMSO only) value, and with >50 events in any double positive gate. **A** and **C** show the proportion of the response accounted for by cells expressing the indicated number of functions. **B** shows the magnitude of the indicated populations in CD4+ T cells, thick bars represent the median and thin bars the interquartile range. SPICE pies were compared using the SPICE permutation test, and dot plots were compared with the Wilcoxon rank sum test. The colours of arcs in **A** and C refer to IFNγ (teal), IL2 (orange), TNF (green) and CD107a (purple).

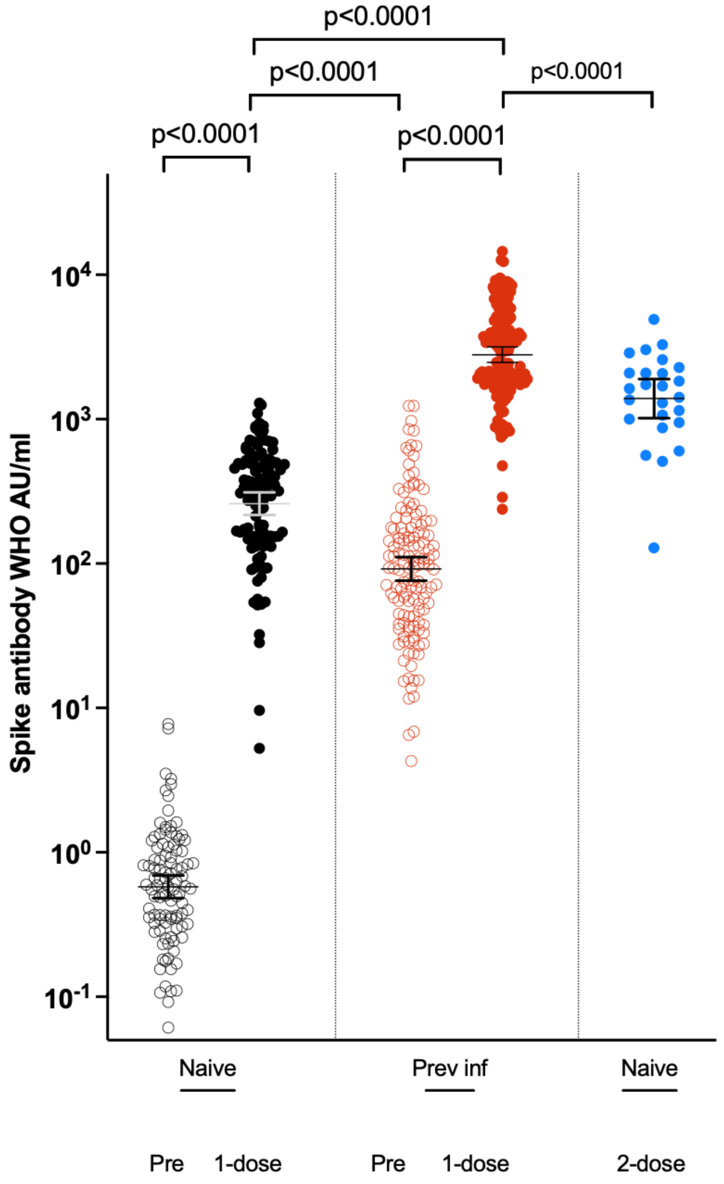

Figure S6. Antibody responses following BNT162b2 vaccine in naïve and previously-infected individuals. (A) Comparison of anti-spike antibody responses (antibody units per millilitre; AU/ml) in naïve (n=111) and previously-infected individuals (Prev Inf, n=142) following a single dose, along with naïve individuals following two doses (2-dose, n=25). Bars represent geometric mean and 95% confidence intervals. Antibody units per millilitre (AU/mL) calibrated to the WHO international standard for anti-SARS-CoV-2 immunoglobulin (NIBSC 20/136). Paired comparisons before and after vaccination were performed using the Wilcoxon matched pairs signed rank test. Unpaired comparisons across multiple groups were performed using the Kruskal-Wallis test with Dunn's post-test for multiple comparisons (adjusted P values displayed). Antibody data are presented on a log10 scaled axis for visualisation with statistical comparisons carried out on untransformed data. Open circles represent responses prior to vaccination. Closed circles represent responses after vaccination.

Table S3. Factors associated with spike-specific antibody response following a single dose of Pfizer BNT162b2 vaccine in a generalised linear regression model (n=253)

Variable	Change in spike-specific T-cell (log10 AU/mL)	Standard Error	*P* value
Previously-infected (vs naive)	+1.05	0.047	<0.0001
Age (in years)	-0.0070	0.0021	0.00086
Sex	-0.0037	0.061	0.95

AU/mL = antibody units per millilitre

Table S4. Factors associated with spike-specific antibody response following a single dose of Pfizer BNT162b2 vaccine in previously infected individuals , in a generalised linear regression model (n=104)

Variable	Change in spike-specific T-cell (log10 AU/mL)	Standard Error	*P* value
Time from SARS-CoV-2 +ve PCR to 1st vaccine dose (days)	+0.0015	0.00033	<0.0001
Age (in years)	-0.0038	0.0024	0.123
Sex	0.014	0.078	0.858

AU/mL = antibody units per millilitre

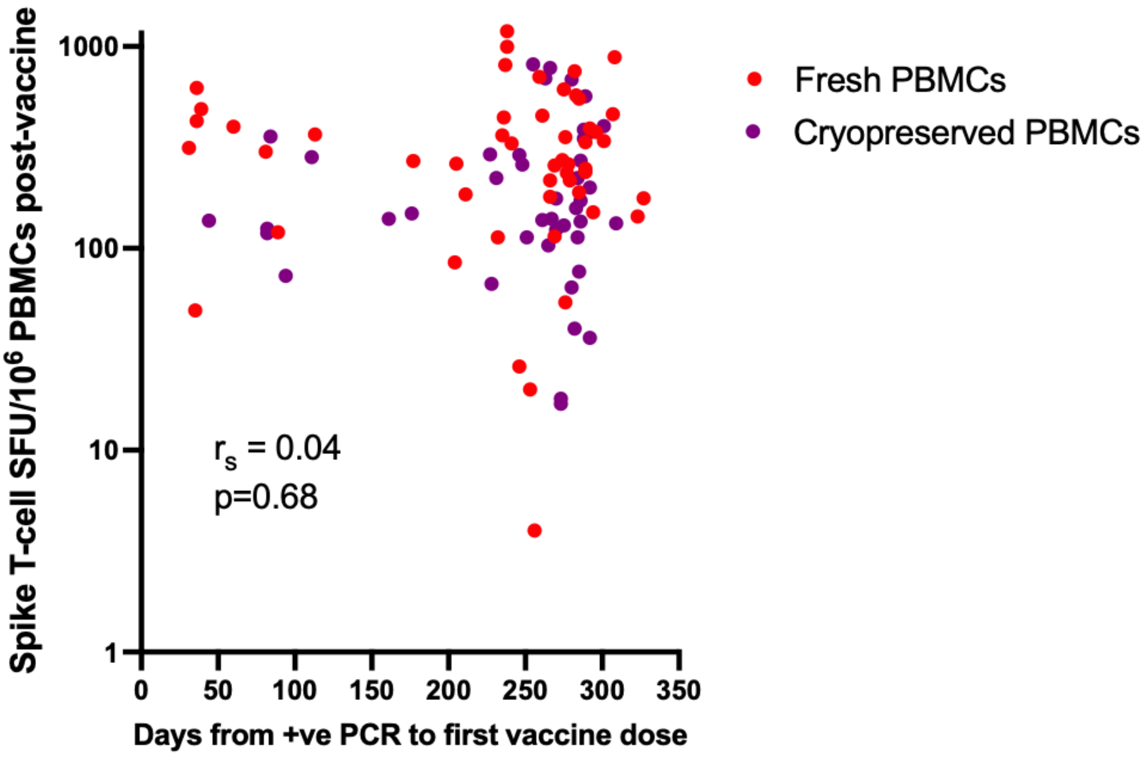

Figure S7. Relationship between time from positive SARS-CoV-2 PCR to 1st vaccine dose and post-vaccine anti-spike T-cell response (Spot forming Units (SFU)/10^6 peripheral blood mononuclear cells (PBMCs)) in previously-infected individuals (n=94). Assays performed using fresh and cryopreserved PBMCs are annotated separately for clarity. Correlation coefficient (Spearman's rho, r_s) and *P* value for whole group is displayed.

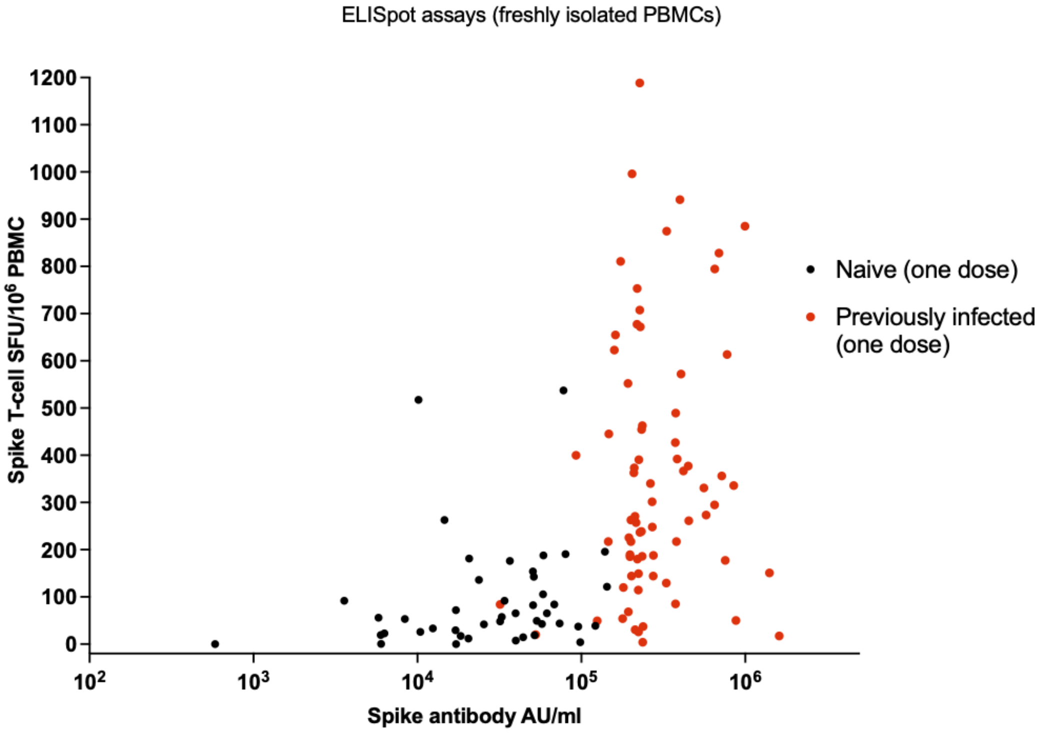

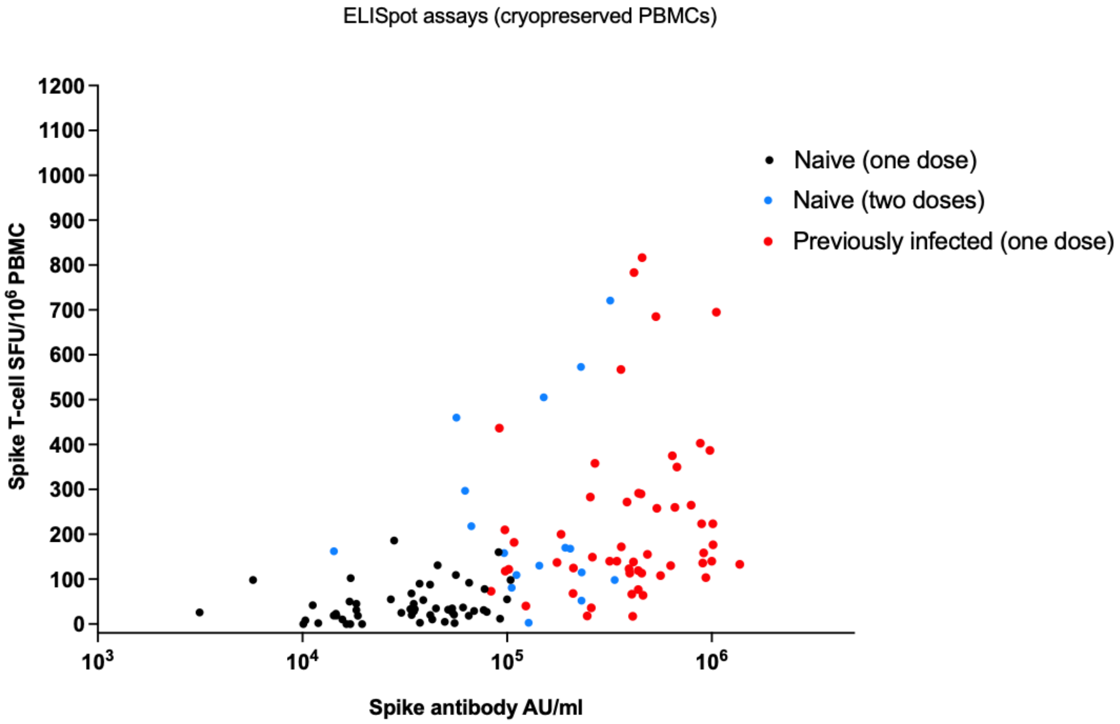

Figure S8. Comparison of anti-spike T-cell responses measured by IFNγ ELISpot (SFU/10^6 PBMC = spot forming units per million peripheral blood mononuclear cells) and anti-spike antibodies (AU/ml) following vaccination. Results stratified by T-cell assays performed on (A) freshly isolated PBMCs and (B) Cryopreserved PBMCs.

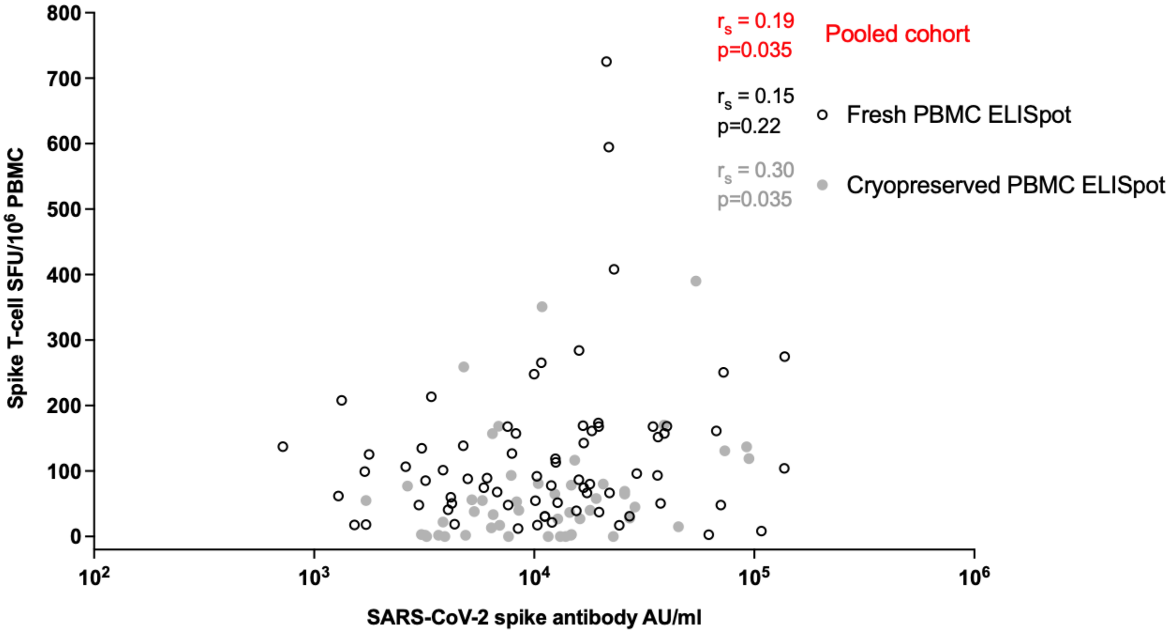

Figure S9. Correlation between antibody and T-cell responses prior to vaccination in previously infected individuals (n=120). Comparison of anti-spike T-cell responses measured by IFNγ ELISpot (SFU/10^6 PBMC = spot forming units per million peripheral blood mononuclear cells) and anti-spike antibodies (AU/ml). Correlation coefficient (Spearman's rho, r_s) of pooled cohort, as well as stratified by T-cell assay (fresh vs cryopreserved PBMCs) is displayed.

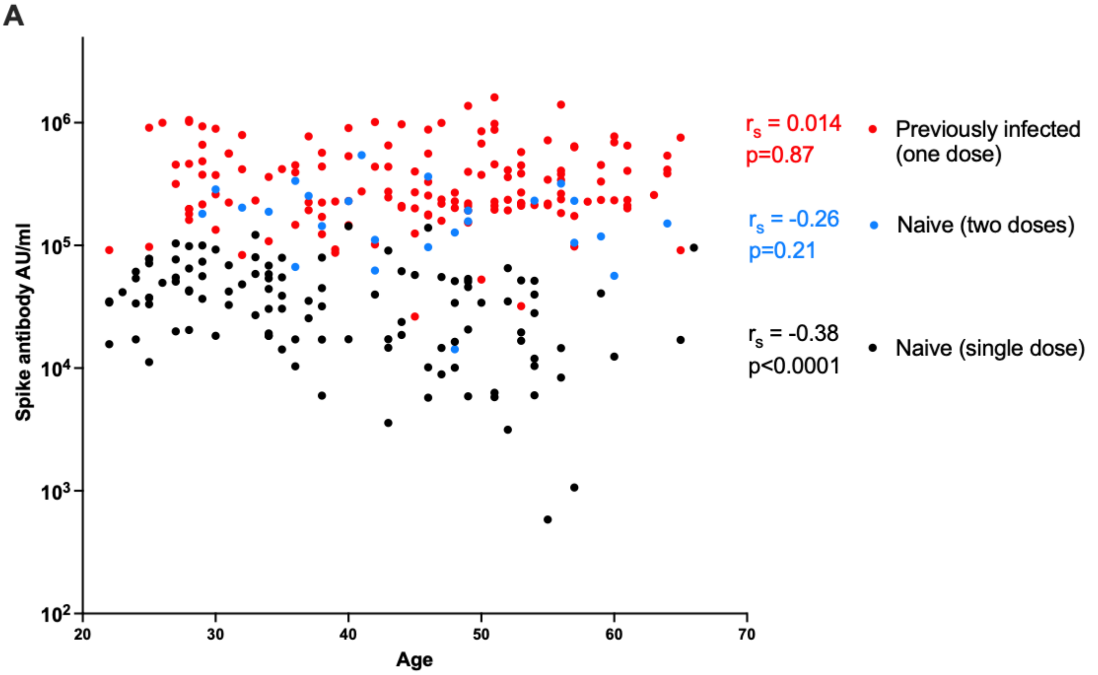

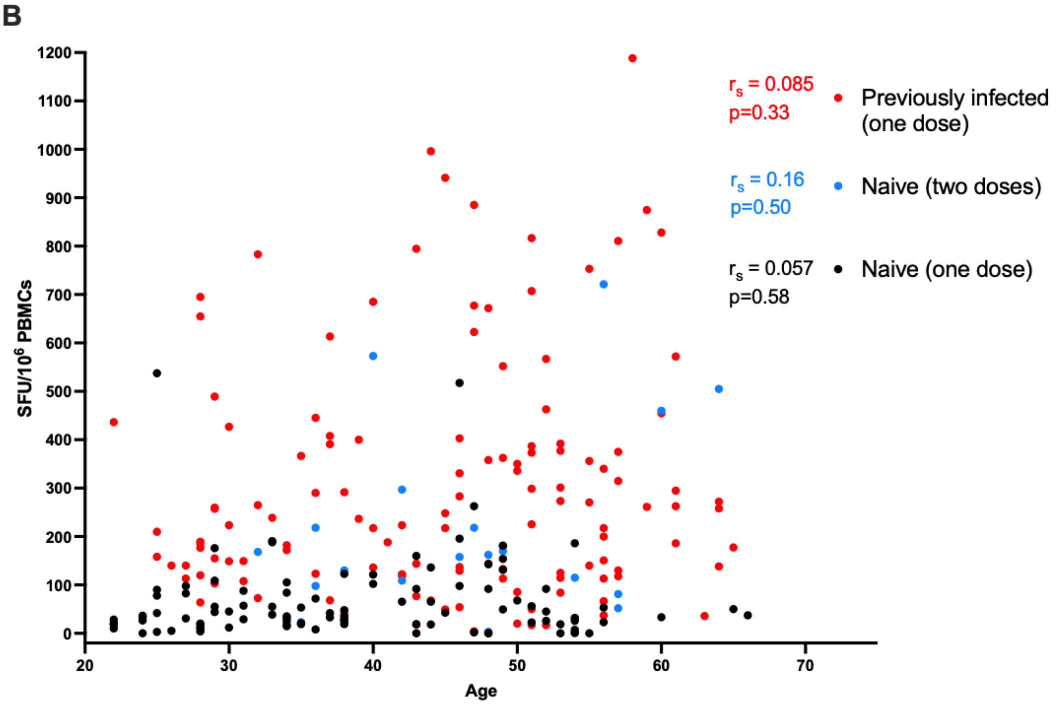

Figure S10. Correlation between age at vaccination and immune responses following BNT162b2 vaccine in naïve and previously infected individuals. (A) Data on anti-spike antibody responses (antibody units per millilitre; AU/ml) in naïve (n=111) and previously-infected individuals (n=142) following a single dose, along with naïve individuals following two doses (n=25). Correlation coefficient (Spearman's rho, r_s) between age and antibody responses displayed separately for the three groups. (B) IFNγ ELISpot responses from combined spike peptide pools in naïve (n=99) and previously-infected (n=130) individuals after a single dose, along with naïve individuals following two doses (n=19). Correlation coefficient (r_s) between age and antibody responses displayed separately for the three groups.

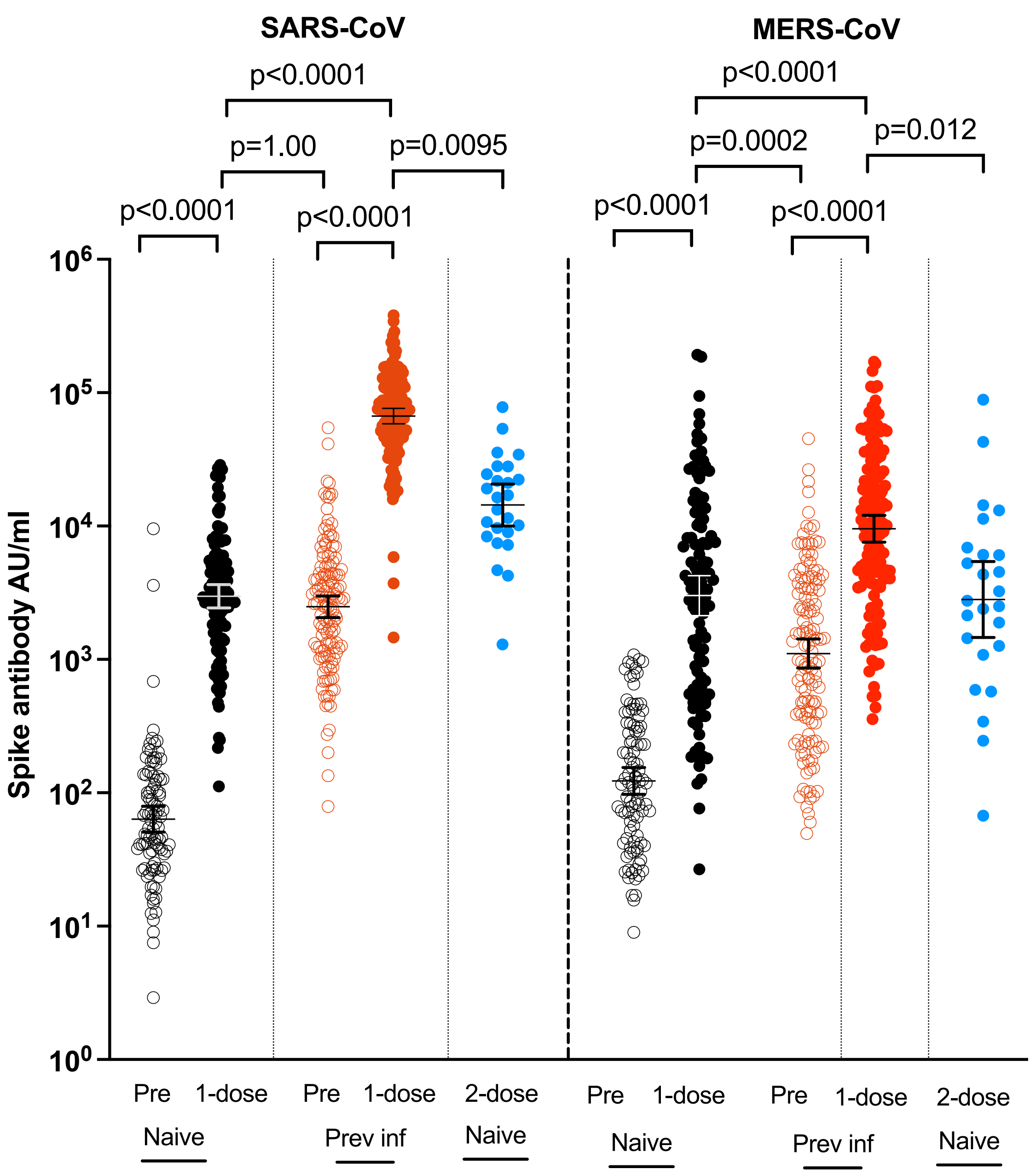

Figure S11. Antibody responses following BNT162b2 vaccine in naïve and previously-infected individuals to SARS-CoV and MERS-CoV. Comparison of SARS-CoV and MERS-CoV anti-spike antibody responses (antibody units per millilitre; AU/ml) in naïve (n=111) and previously-infected individuals (Prev Inf, n=142) following a single dose, along with naïve individuals following two doses (2-dose, n=25). Paired comparisons before and after vaccination were performed using the Wilcoxon matched pairs signed rank test. Unpaired comparisons across multiple groups were performed using the Kruskal-Wallis test with Dunn's post-test for multiple comparisons (adjusted *P* values displayed). Antibody data are presented on a log10 scaled axis for visualisation with statistical comparisons carried out on untransformed data. Open circles represent responses prior to vaccination. Closed circles represent responses after vaccination.

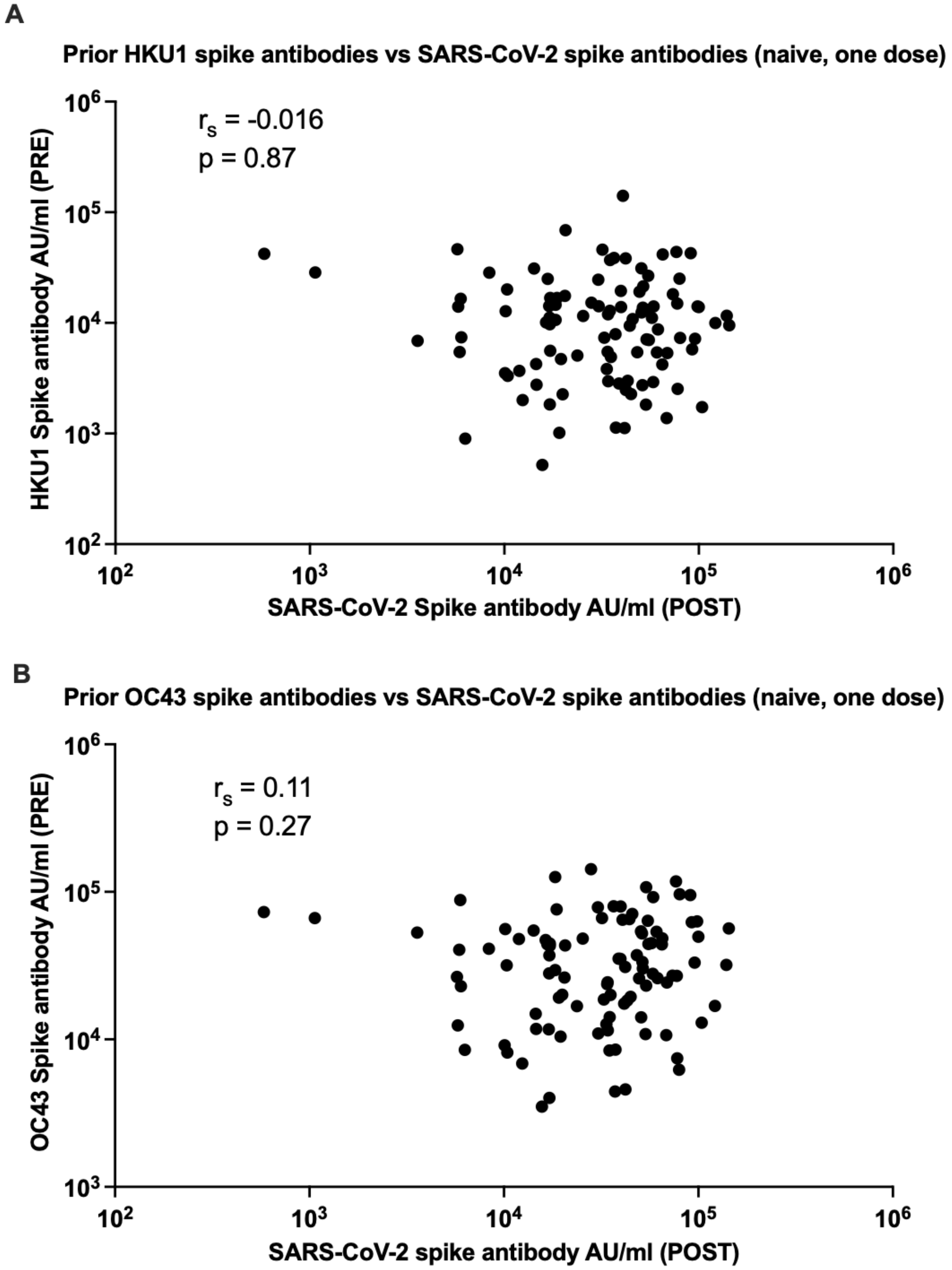

Figure S12. Correlation between pre-vaccine antibodies to seasonal human betacoronavirus spike protein and post-vaccine SARS-CoV-2 antibody response in naïve individuals. Displayed are correlations with (A) HKU1 spike antibody units (AU/ml) and (B) OC43 spike antibody units. Correlation between variables performed using Spearman's rank correlation test (r_s = Spearman's rho). Antibody data are presented on a log10 scaled axis for visualisation with statistical comparisons carried out on untransformed data.

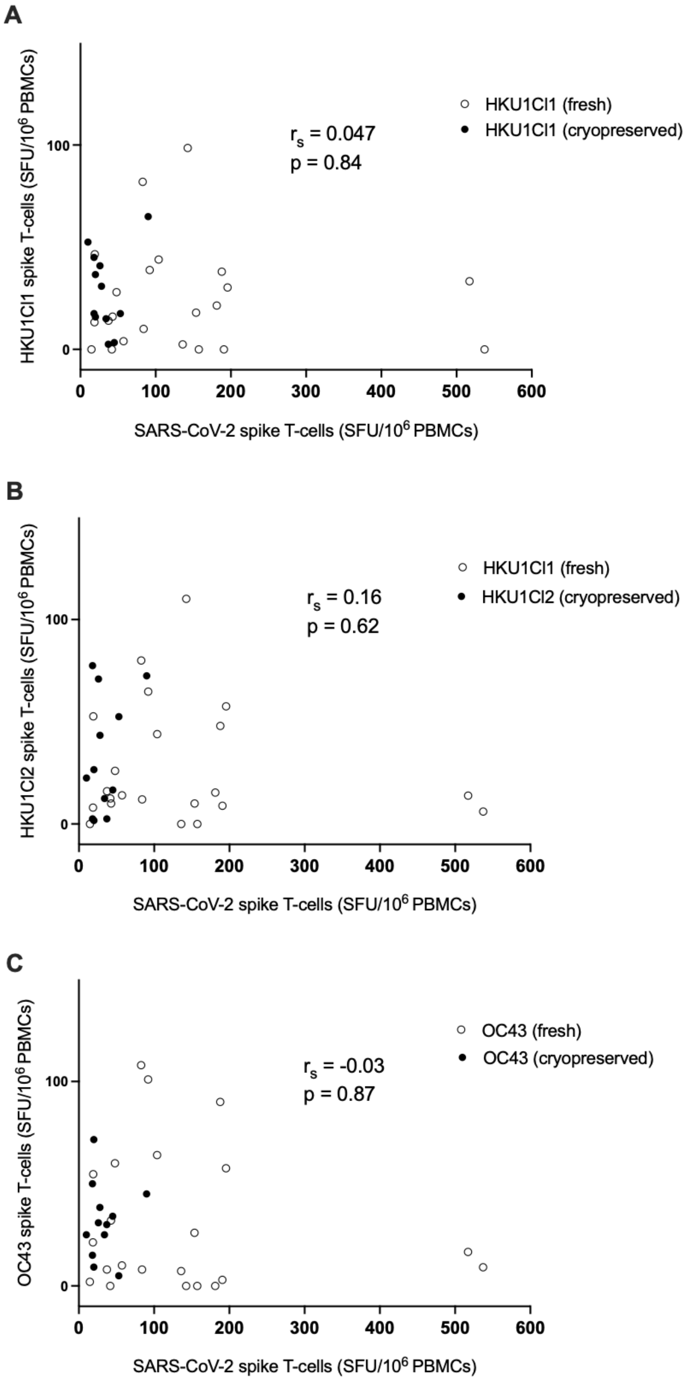

Figure S13. Correlation between pre-vaccine T-cell responses to seasonal human betacoronavirus spike protein and post-vaccine SARS-CoV-2 spike T-cell responses in naïve individuals. IFNγ ELISpot responses from n=35 individuals expressed as spot forming units per million peripheral blood mononuclear cells (PBMCs). Responses to seasonal coronaviruses are summed data from overlapping peptide pools representing S1 and S2 spike subunits to (A) HKU1 clade 1 (HKU1Cl1) (B) HKU1 clade 2 (HKU1Cl2) and (C) OC43. All seasonal coronavirus data are generated using cryopreserved PBMCs, whereas post-vaccine SARS-CoV-2 spike responses were from both freshly isolated (open circles) and cryopreserved (closed circles) PBMCs. Correlation between variables performed using Spearman's rank correlation test (r_s = Spearman's rho).

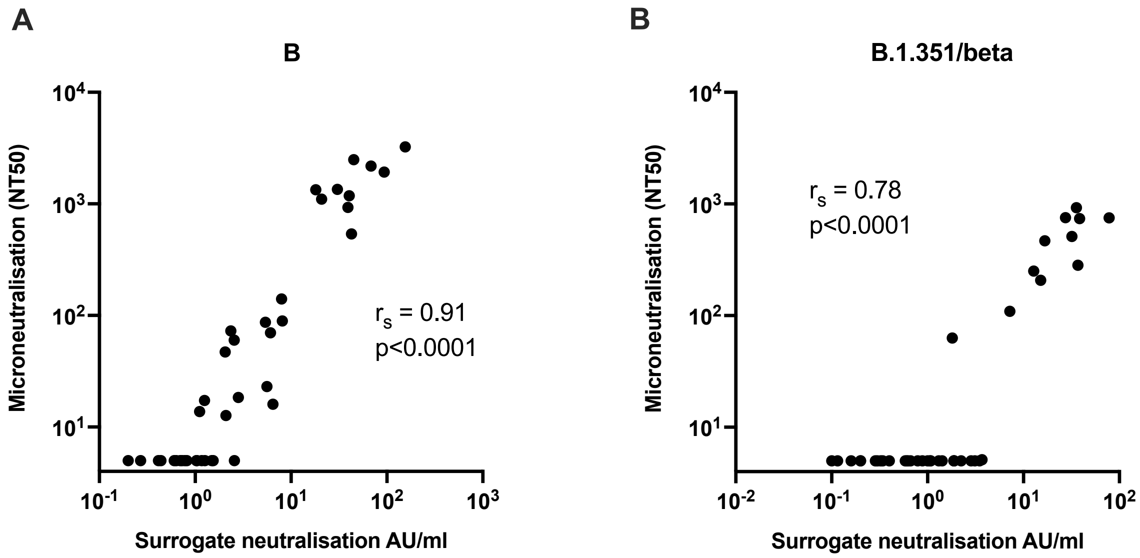

Figure S14. Correlation between live virus neutralisation and surrogate neutralisation assay for Wuhan-1 like (B lineage) and B.1.351/beta lineage SARS-CoV-2. Surrogate neutralisation activity before and after one vaccine dose in naïve (n=10) and previously-infected (Prev Inf, n=10) individuals to spike proteins from (A) Wuhan-1 like B lineage virus and (B) B.1.351/beta lineage, expressed as antibody units (AU/ml), compared to ability of same plasma to neutralise live virus, expressed as the reciprocal titre required for 50% reduction in infectious focus-forming units (NT50) of lineage B and B.1.351/beta viruses in a microneutralisation assay. Correlation between variables performed using Spearman's rank correlation test.

Table S5. Surrogate neutralisation activity (units/mL) against different SARS-CoV-2 variants before and after one vaccine dose in SARS-CoV-2 naïve and SARS-CoV-2 previously infected individuals

	**SARS-CoV-2 naïve (n=10 individuals)**							
	D614G Pre-dose	D614G Post-dose	B.1.1.7/alpha Pre-dose	B.1.1.7/alpha Post-dose	B.1.351/beta Pre-dose	B.1.351/beta Post-dose	P.1/gamma Pre-dose	P.1/gamma Post-dose
**Median**	0.575	5.34	0.4	3.07	0.465	1.89	0.33	1.48
**25% Percentile**	0.445	3.378	0.2	1.85	0.2475	1.393	0.2	1.038
**75% Percentile**	0.695	8.393	0.665	5.098	0.8125	3.203	0.5075	2.94
	**SARS-CoV-2 previously infected (n=10 individuals)**							
	D614G Pre-dose	D614G Post-dose	B.1.1.7/alpha Pre-dose	B.1.1.7/alpha Post-dose	B.1.351/beta Pre-dose	B.1.351/beta Post-dose	P.1/gamma Pre-dose	P.1/gamma Post-dose
**Median**	1.52	65.93	0.905	34.7	0.495	29.72	0.435	22.64
**25% Percentile**	0.885	52.08	0.475	25.79	0.2	14.55	0.2	14.78
**75% Percentile**	3.075	121.5	1.538	61.77	1.263	37.35	1.19	32.13

PITCH_SOP_001

SOP Title: ELISpot assay using Mabtech IFN-γ Kit

1.0	**Purpose/ Introduction:**

This protocol provides the procedure for performing an *ex vivo* ELISpot assay using frozen peripheral blood mononuclear cells from human blood of patients with COVID19 (PCR confirmed or syndrome) as well as COVID19 recovered.

The principle of an ELISpot assay is similar to an ELISA. However, the assay does not aim in quantifying the concentration of a certain cytokine in cell culture supernatants/serum as it is done in ELISA. ELISpot aims in visualizing and further quantifying the number of cells in a sample, which secrete a certain cytokine.
First a capture antibody is bound to a PVDF membrane in a 96 well plate. After several washes and blocking the membrane with bovine serum albumin, cells are plated into the wells and treated as desired (1.). If cells secrete the cytokine of interest, it will be bound by the capture antibody on the membrane (2). After incubation of usually 24-48 hours (depending on the cytokine measured) at 37C, 5% CO_2 and 95% humidity, the cells are washed off and the plates are incubated with biotinylated detection antibody which will bind to the cytokines captured on the membrane (3). To detect the antibody-cytokine-antibody+biotin complex streptavidin conjugated with alkaline phosphatase (AP) is typically added to the wells (4). In the final step BCIP/NBT will be added to wells which acts as substrate for the enzyme AP (5). AP catalyzes the hydrolysis of phosphate groups from the substrate molecule, resulting in a coloured product (i.e. blue).

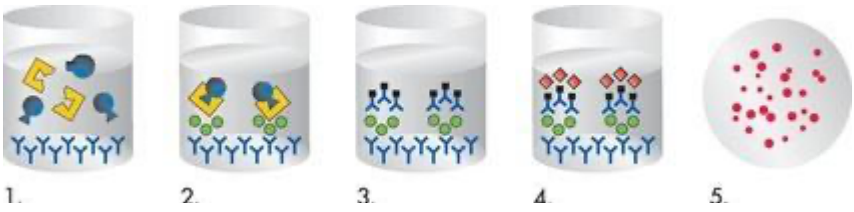

2.0	**Scope / Responsibility:**
	Provide a list as to which specific departments or designees this procedure will apply to.

Local laboratory staff working on PITCH, PHOSP, ISARIC4C, OCTAVE, ALSPAC studies across all sites.

3.0	**Definitions:**
	Provide definitions for all important words and abbreviations referenced in this procedure.

FCS	Fetal calf serum
ELISpot	Enzyme linked immunosorbent spot
PBMC	Peripheral blood mononuclear cells
PBS	Phosphate buffered saline
PS	Penicillin/streptomycin
RPMI	Cell culture medium with bicarbonate buffering system, amino acids and vitamins
RT	Room temperature
CEF virus)	CD8 Peptide pool (Cytomegalovirus, Eppstein Barr virus, Influenza
PHA	Phytohemagglutinin-L (non-specific positive control, activates T-cells)

4.0	Associated Standard Forms:
	List all forms associated with this procedure.<br><br>Refer to local documents.

5.0 Responsibilities

Responsibility for supervising the use of this protocol by laboratory staff, in accordance with biosafety requirements is determined locally. **This protocol uses human blood. All users MUST have health and safety clearance.**

6.0 Reagents and Materials

Item	Suggested Catalog No.	Suggested Supplier	Notes
L-Glutamine	G7513	Merck Life Science UK Limited, Gilingham, Dorset, UK	
RPMI-1640 Medium with Sodium bicarbonate, no L-Glutamine	R0883	Merck Life Science UK Limited, Gilingham, Dorset, UK	
Penicillin/Streptomycin	P0781	Merck Life Science UK Limited, Gilingham, Dorset, UK	
Fetal Calf Serum heat inactivated	F9665-500ML	Merck Life Science UK Limited, Gilingham, Dorset, UK	Alternatives may be used but these must be heat inactivated, sterile and suitable for cell culture
Carbonate/bicarbonate capsules	C3041-100CAP	Merck Life Science UK Limited,	

		Gilingham, Dorset, UK	
CEF (FEC) peptides		Proimmune, Oxford, UK	
Phytohemagglutinin-L	11249738001	Merck Life Science UK Limited, Gilingham, Dorset, UK	
Phosphate buffered saline tablets	12821680	Fisher Scientific, Loughborough, UK	
Tween 20	P2287-500ml	Merck Life Science UK Limited, Gilingham, Dorset, UK	
IFN-γ human ELISpot antibody clone 1-D1K	3420-3-1000	Mabtech AB, Nacka Strand, Sweden	Alternatively, can be purchased as kit
IFN-γ human biotinylated antibody clone 7-B6-1	3420-6-1000	Mabtech AB, Nacka Strand, Sweden	Alternatively, can be purchased as kit
Streptavidin ALP for ELISpot	3310-10-1000	Mabech AB, Nacka Strand, Sweden	Alternatively, can be purchased as kit
Multiscreen-I 96 ELISpot plates	MAIPS4510	Millipore, Watford, Hertfordshire, UK	Alternatives may be used by they must have PVDF membrane.
BCIP/NBT	1706432	BioRad, Watford, Hertfordshire, UK	

Solutions to be made up

R0

- 500 ml RPMI 1640 (without glutamine)
- 5 ml 10000 U/ml penicillin/streptomycin from aliquot.
- 5 ml 200 mM L-Glutamine from aliquot.
- Label “R0” and write date, initials on the bottle. Store at 4°C and replace after 1 month or if any concern about sterility

R10

- 500ml RPMI 1640 (without glutamine)
- 5 ml 10000 U/ml penicillin/streptomycin from aliquot
- 5ml 200 mM L-Glutamine from aliquot
- Add 50ml filtered heat inactivated FCS. Keep a note of the FCS lot number.

- Label “R10” and write date, initials on the bottle. Store at 4C and replace after 1 month or if any concern about sterility

PBS

- Made with PBS tablets and dH2O. 1 tablet in 100ml water.
- Autoclave and store at RT and keep sterile.

ELISpot coating buffer

- Dissolve contents of one carbonate-bicarbonate buffer capsule (Merck Life Science UK Limited, Gilingham, Dorset, UK) in 100 ml deionized water. (the powder only, do not add the plastic casing)
- Autoclave and store at RT and keep sterile.

PBS-Tween 0.05%

- PBS bottles must be sterilised.
- Using a Pasteur pipette add 250µl of Tween 20 to a 500ml bottle or 500µl to a 1 litre bottle of PBS
- Replace lid tightly and invert several times to mix
- Label bottle as ELISpot wash buffer with date made up

SARS-CoV2 Peptides @4ug/ml for final concentration of 2ug/ml

A. Mimitopes 15mer peptide pools: S1, S2, M+N
B. Controls: R10, PHA, CEF

S1, S2 and R10 will be assayed in triplicate and M+N, CEF, PHA in duplicate. Core assay of 15 wells requires 3 million cells. NSP3b (mimotopes library peptides 202-291) to be added to core assay if there are enough cells available.
All peptides are made up to 2x final concentration for direct use in ELISpot assays.
Please remove peptides from the -80 freezer AFTER cell counts have been assessed to avoid wastage. Please refer to assay priority document for allocation of cells to different assays.
If any significant volume of peptide is left over after setting up assays, leave in fridge for use the following day.

Samples
PBMC cell suspension: adjusted to 4x10^6 c/ml in R10.

7.0 Procedure

Safety Measures

Please refer to local safety procedures and risk assessments.

Coating of plates (d-1 or d0)

- Prepare the coating solution by adding 10 µl of capture antibody (1-D1K- green lid) per ml of ELISpot coating buffer resulting in a final concentration of 10 µg/ml

Ab. You will need 0.75 ml of coating solution per subject for core assay (15 wells per subject), but it is a good idea to prepare a little more to account for pipetting errors if doing lots of plates. Make sure you have the correct MabTech kit/Ab.

- Label ELISpot plate(s) with date, initials, experiment ID (e.g. SITE_PITCH_E00x). Make sure A1 is at the top left. Please label both lid and base of plate to avoid mix-ups.
- Add 50µl per well of the coating solution to the ELISpot plate with a multi-channel pipette.
- Tap the plate gently to ensure that all the wells are completely covered by the coating buffer. Check all wells are covered.
- Wrap the plate in cling film and either keep at room temperature (RT) for 3-8h or at +4C (fridge) for 8-48hs.

Assay setup (d0)

BLOCKING THE PLATE

- Remove the coating solution into a plastic box (in the hood), and wash the plates 2 times by adding 100 µl sterile PBS or R0 to each well using a multichannel pipette and removing the washing solution.
- Block the wells by adding 200 µl R10 to each well with a multichannel pipette. Keep the plate at RT for 1/2h-8h or alternatively at +4C for 8-48h.

ADDING CELLS TO THE ELISPOT PLATE

For each study use a similar layout. This makes data analysis and spot trouble shooting much simpler. Label the plate clearly and cross out any well that were not used.

- Remove the blocking solution from the ELISPOT plate (into a tissue lined plastic box in the hood).
- Add 50µl of 2x concentrated stimulants to the test wells.
- Add 50µl of 2x concentrated PHA, CEF to your positive control wells, add 50µl of R10 to your negative control wells. There should be positive and negative control wells on each plate.
- Add 50µl of cell suspension (200,000 cells from a 4x10^6 c/ml suspension in R10) to the appropriate wells on the plate. Prepare duplicate wells per stimulation condition per sample.
- Place the plate in a suitable container.
- Incubate the plate(s) for 16-18 hs at 37C, 5% CO2, 95% humidity in the tissue culture incubator.

Assay Development (d1)

DETECTION

- Carefully take off cell suspension with multichannel pipette, place into a fresh 96 well plate and freeze for future use (-80C, label with Patient ID, Experiment ID, date and initials).

- Using the multi-channel pipette, wash 6 times by adding 200 µl PBS-Tween to each well. Between each wash take off the washing solution. Make sure not to scratch the well surface with the pipette tip.
- Dilute the detection antibody (7-B6-1-Biotin-Yellow Lid) 1:1000 in PBS resulting in a concentration of 1ug/ml, and add 50µl to each well
- Incubate the plate for 2-4 hs at RT or overnight at 4C in the fridge (within a secondary container)
- Take off the detector antibody and using a multi-channel pipette, wash 6 times by adding 200µl PBS-Tween to each well. Between each wash, take off washing solution.
- Dilute the streptavidin alkaline phosphatase antibody (Mabtech) 1:1000 in PBS, and add 50µl to each well.
- Incubate for 1-2hs at RT.
- Remove the bottle of BCIP/NBT stock solution from fridge and place at RT
- At the end of incubation take off the SA-ALP solution and, using a multi-channel pipette, wash 6 times with PBS-Tween. Take off the washing solution.

DEVELOPMENT

- Invert the bottle of BCIP/NBT solution at least five times to mix. The bottle should be approximately at room temperature.
- Transfer 6 ml BCIP/NBT solution per plate to an appropriate Falcon tube or bottle with a 0.22 µm filter.
- Dispense pre-filtered BCIP/NBT solution into a sterile reagent reservoir and transfer 50 µl to each well of every plate.
- Incubate the plate(s) at room temperature for 5-7 minutes, or until distinct dark spots appear in the positive control wells.
- To stop colour development, decant the BCIP/NBT solution and rinse each plate three times with tap water. Remove the rubber bottom from all plates and rinse the bottom side of the membrane with tap water.
- Allow to air-dry overnight.

COUNTING THE ELISPOT PLATE

- Wait a minimum of 2 days before counting the plates to allow the membrane to dry completely
- Once dry read the plates on the ELISpot Reader available at site.
- Scan the plate lids. Upload the Excel plate count, the PDF plate scan and the Image file Plate to the appropriate results folder
- After reading the plates store them in aluminium foil in a dark dry space

8.0 Risk Assessment

See local risk assessments specific to sites.
